# Ethnoveterinary medicinal plants and their utilization by the people of Soro District, Hadiya Zone, southern Ethiopia

**DOI:** 10.1186/s13002-024-00651-6

**Published:** 2024-02-22

**Authors:** Mulatu Hankiso, Zemede Asfaw, Bikila Warkineh, Abiy Abebe, Bihonegn Sisay, Asfaw Debella

**Affiliations:** 1 Department of Plant Biology and Biodiversity Management, College of Natural and Computational Sciences, Addis Ababa University, P.O. Box 1176, Addis Ababa, Ethiopia; 2 Biology Department of Natural Science, Hossana College of Education, P.O. Box 94, Hossana, Ethiopia; 3 Ethiopian Public Health Institute, P.O. Box 1242/5654, Addis Ababa, Ethiopia

**Keywords:** Ethnoveterinary medicine, Herbal drug knowledge, Livestock ailments, Medicinal plants

## Abstract

**Background:**

Ethnoveterinary studies are important to maintain the sustainability of livestock health and support people’s livelihoods through the provision of food, maintaining livestock health, and other biological resources. This study was carried out in Soro District, southern Ethiopia, to identify, document and analyse plant species with ethnoveterinary uses along with the associated indigenous and local knowledge.

**Materials and methods:**

Informants were selected using purposive (key informants) and systematic random sampling (general informants) methods. Data on ethnoveterinary plants and their uses were collected through semi-structured interviews, guided field walks, 13 focus group discussions with five to seven members in each and participant observation. Informant consensus factor and fidelity level were computed to identify the most common livestock ailment categories and the best plant species with ethnoveterinary use, respectively. Preference ranking methods were used to identify the potentially effective ethnoveterinary medicinal plants for the most frequently reported livestock ailments. The use diversity of multipurpose plants with ethnoveterinary importance was analysed using the analytical methods of ethnobotany including priority ranking, comparisons and important indices. The T-test statistic was used to compare knowledge differences among different social groups.

**Results:**

A total of 132 plant species in 120 genera and 61 families were reported by informants as having ethnoveterinary uses. The plants are said to be used by the local communities in various ways to treat 50 livestock health problems. Higher number of informants (23.77%) cited *Momordica foetida* for the treatment of 16 livestock ailments. The highest informant consensus value for this species is associated with its use for treating blackleg in cattle; *Nicotiana tabacum* was cited for the treatment of 15 livestock ailments mainly recommended for the Lumpy Skin Disease/Ailment of bovines; *Croton macrostachyus* for treatment of 13 livestock ailments including wooden tongue, FMD in bovines; and *Gymnanthemum amygdalinum* for nine ailments mainly diarrhoea of all livestock types. *Achyranthes aspera* is claimed to provide the most effective treatment for *Aspiration pneumonia* (severe coughing in bovines, sheep and goats) alone, while *Croton macrostachyus, Ximenia americana*, *Allium sativum* and *Juniperus procera* were indicated as potential plant species to treat Lumpy Skin Disease in bovines in the order given. The fidelity level analysis showed that *Datura* stramonium*, Dodonaea viscosa* subsp*. angustifolia* and *Asparagus africanus* were potential medicinal plant species to treat the respective ailments of rabies, Peste des petits ruminants (PPR) and evil eye/spirit. Multipurpose plant species including *Prunus africanus, Combretum molle* and *Afrocarpus falcatus* have been highly threatened as indicated by direct matrix ranking mainly due to collection of fuel wood, construction materials and making household utensils, and farm implements rather than for other uses.

**Conclusion:**

Soro District has rich and diversified livestock herbal medicinal resources, and indigenous knowledge of remedy preparations and applications is transmitted through generation lines. This resource faces anthropogenic threats with deforestation being the leading factor. Consequently, ethnoveterinary medicinal plants continue to decline before adequate and proper scientific documentation and testing are made. There is a dire need for planning and implementation of appropriate in situ and ex situ conservation strategies and to strive towards ensuring the survival and sustainable utilization of such important plant resources of Soro District. This must be supported by further documentation of the associated indigenous knowledge and pharmacological testing of the key promising species including *Balanites aegyptiaca (*novel species/NS to treat specific ailment)*, Brugmansia suaveolens* (novel species/NS reported first to treat Livestock ailments/LsAs)*, Euclea divinorum* (NS to treat specific ailments), *Grevillea robusta* (NS), *Hagenia abyssinica* (NS for the reported specific ailment)*, Pentanema confertiflorum* (NS*), Juniperus procera* (NS), *Maesa lanceolata* (NS)*, Millettia ferruginea* (NS for reported specific ailments)*, Schrebera alata*/NS*, Securidaca longepedunculata*, *Spiniluma oxyacantha*/NS, *Vepris nobilis* (novel species reported first to treat LsAs), *Zanthoxylum asiaticum* /NS and *Ximenia americana* (NS for specific ailments). This ethnoveterinary study attempted to fill part of the gaps concerning the prevalent livestock health problems and the associated indigenous and local knowledge in the area.

## Background

Ethnobotany of livestock medicinal plants is concerned with the study of the intimate association between the plants and the people and is encoded in the indigenous and local knowledge and practices that went on deepening and enhancing through human generations. This body of knowledge system needs further enhancement before the knowledgeable elderly people of the community pass away; the social fabric is transformed and the environment changes in one way or the other along with the decline of the useful plant resources. An exhaustive investigation of traditional knowledge in ethnoveterinary herbal medicine with the cooperation between herbalists, ethnobiologists, veterinary scientists and anthropologists can continue to move forward through integration and intimate relations with modern veterinary medicine. Such collaboration and cooperation among the key stakeholders helps to engage the society and governmental institutions [1]. In addition, higher proportion of the African people about 80% of the population [2, 3] or more use potential medicinal plants for treatment of various livestock ailments. In Ethiopia, ethnoveterinary medicinal practices using medicinal plants are alternative options to cure more than 90% of the country’s huge livestock population, and also for more than 80% human population [4, 5]. Furthermore, plant remedies are used against livestock health retribution in more proportion [6–8]. Although modern veterinary services have been there for a long period [9], traditional herbal medicine has been repeatedly and increasingly shown to have effective healing power for a number of livestock ailments. Moreover, Ethiopian traditional veterinary practitioners contribute to the welfare of domestic animals, their productions and management [10].

Limited distribution of modern veterinary healthcare services, unaffordable cost and lack of accessibility to healthcare benefits makes herb-based ethnoveterinary practices mandatory [11]. Thus, to cover the gap in healthcare service through centuries in developing countries of the world, farmers and pastoralist in rural communities have been depending on the wealth of traditional medicines to manage livestock ailments [11], to increase their productive yield and most of this is achieved through herbal medicine; various livestock ailments, pathogenies and vectors are the major constraints that decrease domestic animal production and development in different marginal and rural areas of Africa [12]. Traditional herbal medicine provides a safeguard for the group of domestic livestock such as bovines (cattle), equines (horses, donkeys and mules), goats, sheep including poultry (chickens/hens) and is directly related to the food security, and to the sources of economic income due to the systems of healthcare [9]. However, the treatment of livestock ailments using traditional herbal medicine had begun before the formulation of modern drugs as reported from different countries [8]. Nowadays, the use of the ethnoveterinary medicinal plants along with the associated indigenous knowledge is transferred among successive generations of people only orally without written records and no adequate scientific documentation exists [13]. The same source explained that the transfer of this herbal medicinal heritage system is heading to deterioration and decline and may even lead to the eventual extinction of the indigenous medicinal plants, which also leads to impacts on food security as well as negative impacts on ecological transition.

The role of veterinary practices to treat livestock ailments is a long-time practice in all parts of the world, especially in developing countries where livestock healthcare facilities and services are still very few and located scarcely at urban centres [14]. Even those people living in close proximity to areas where modern drugs/pharmaceutical products are readily available use preparations of traditional medicinal plants to treat their domestic livestock. This is related to shortage of modern drugs, cultural acceptability, relative efficacy in fighting certain ailments and economic affordability for the rural communities [15]. Furthermore, the use of traditional plant-based medicines fits well with the necessity of healthcare system and management of different multifunctional livestock, but the knowledge of veterinary practice is declining as the plants become less and less in their local habitats due to many threatening factors. The consequence is the decline of food serving domestic livestock and other multipurpose livestock, wild forage plants and ecological services. The anthropogenic activity of deforestation for expansions of settlement areas, farming lands, rearing livestock, overexploitation of plants for various other purposes lead to environmental degradation and to threats of medicinal plants [4, 5], which may in turn lead to the loss of livestock lives.

Indigenous knowledge on livestock herbal medicine and practices is being transmitted to the young generation via oral message rather than in the form of written documents and stories [7]. As a result, veterinary traditions went on eroding without adequate documentation based on suitable and effective analysis of medicinal plant alongside the associated indigenous knowledge [11]. Soro District, an area of agriculturists and agropastoralists where mixed agriculture is widely practised, has long been inhabited by people who have a long tradition of using medicinal plants to treat livestock ailments and there has been very little effort to assess and document ethnoveterinary plants and associated knowledge and practice. Given the absence of livestock medicinal plant studies so far in Soro District, a strategic plan was made and this study is an initiative to assess and document ethnomedicinal plants of veterinary importance with the associated uses and local practices particularly focussing on Soro District of Hadiya Zone.

Moreover, in different phytogeographical regions of East African countries, many studies in various areas of Ethiopia with diverse ethnic groups and biodiversity have also documented ethnoveterinary healthcare of livestock with medicinal plants along with the associated traditional knowledge. In addition, solely ethnoveterinary study in the Soro District also contributes rich sources of livestock medicinal plants with traditional knowledge, which also provide wild food and ecologically important plants. Thus, the objective of this study was to document the diversity of livestock medicinal plants paying special regard to fill the information gap on ethnoveterinary plants and their associated functions that enhance livestock welfare and food security. This indigenous knowledge documentation and transfer to future generations could determine the status of livestock, their health management system and their threats in the study area and beyond. Therefore, the study also planned to assess and document ethnoveterinary medicinal plants and associated indigenous knowledge and traditional practices in Soro District, Hadiya Zone, southern Ethiopia.

## Methods

### Study area description

The ethnobotanical study was conducted in Soro District, geographically located between the coordinates 37^0^ 20′ 0’’ to 37^0^ 50′ 0’’E longitudes and 07^0^ 0′ 0’’ to 07^0^ 40′ 0’’N latitudes; and the altitude ranged from 799 m.a.s.l to 2934 m.a.s.l. The total land area covers 36,473.337 km^2^/3647333.7 ha. Most of the study District sites are highlands followed by middle and low land agroclimatic zones, and the mean annual rain fall is in between 900 and 1500 mm, with the temperature in between 12 and 26 °C.

The study area, Soro District, was selected purposively by the researcher, whereas sampled kebeles were chosen by a focus group discussion conducted at the district level at the beginning of the research when reconnaissance study was conducted. Soro District is grouped as one of the high agricultural potential areas in Hadiya Zone, and the main economic activity is agriculture. The main interest in this study is to document the ethnobotanical information with the associated indigenous knowledge focussing in this study on livestock ailments and the medicinal plants used by the local community to manage these ailments. The total human population of Soro District is 287, 589; with 143, 835 males and 143, 754 females. The majority (about 87.42%) of the people live in rural communities who mainly rely on agricultural economy and 12.58% of the people live in urban areas. The indigenous people of Soro inhabiting Soro District belong to Hadiya people, who speak the Cushitic family language in Ethiopia (which is one of the major ethnolinguistic groups in Ethiopia). They speak the language of Hadiyissa and learn it in the school where it is part of the formal education of the school-aged children; they also use Amharic (the national language) for official work.

The livestock population in the District as given by Soro District Agricultural and Rural Development Office in 2020 the livestock population of the District has been estimated to be about 3, 329, 827, of which the highest proportion, about 29.62%, goes to herds of bovines (cattle), accounting for 986, 248, followed by 388,082 sheep (11.5%), 295,018 goats (8.86%), 40,291 equines, 1, 620, 188 poultry including 18, 918 honeybee colonies and 0.5 tone fish. These major agricultural commodities indicate the type of agricultural activities and that the communities are more of semi-pastoralists and agropastoralists that keep very high livestock population. However, in spite of the presence of high livestock population there are only few numbers of veterinary clinics in the District and most households rely on traditional herbal medicine to treat different kinds of livestock ailments.

The Head of Veterinary Office in the District reported that Lumpy Skin Disease (LSD), blackleg, trypanomiasis, Foot and Mouth Disease (FMD), PPR, New Castle Disease (NCD), cattle pasteurellosis, African Horse Sickness (AHS), rabies, livestock tuberculosis, anthrax, shoat pox, fowl typhoid, coccidiosis, livestock lice/tick infection and other parasitic diseases were among the most common ailments frequently affecting livestock in the District. There are 14 rural veterinary healthcare posts and one main clinic in the main town of the District. The rest of the 18 administrative kebele’s get veterinary service from nearby health posts and a clinic. There were eight veterinarians; three DVMs, five  Bsc and 15 diploma holders without any livestock healthcare assistants working in the District. The mentioned veterinary healthcare centres are not sufficient to provide proper health services for the total number of livestock heads present in the study area. This was due to the migration of veterinarians, health clinics and professionals. Health services were more or less used in clusters.

Furthermore, the rising human population and expansion of farming are the main contributing factors to the declining vegetation of the study area. This was the key factor that motivated initiation of this research that investigated ethnoveterinary medicinal plants and the associated community knowledge of the people of Soro District.

Based on the information gathered during the reconnaissance survey and archival sources [16] at the District level, the research was informed that there are 33 total kebeles, of which 13 rural kebeles/subdistricts (the smallest administrative units) or resident sites including nearby urban kebeles were purposively selected and involved in this research. The map of the study District and the selected sites are as given in our recent publication on the wild edible plants of Soro District [17].

### Focus group discussions

A focus group discussion in the centre of the District (Gimbichu town) consisted of different members of key stakeholders from Soro District administrative offices representing relevant professions and social groups, including health, veterinary and fisheries, culture and tourism, agriculture, education, environmental protection, forest and climate change, biodiversity, public service (capacity building), children, women and youth offices. During actual data collection, 13 focus group discussions were conducted, in the entire research area with the collaboration of different professionals, and semi-structured interviews of purposively sampled key and systematic randomly sampled general informants.

In a focus group discussion, 12 informants (eight males and four females) were involved in Gimbichu town. One FGD was conducted at each data collection site using semi-structured interviews with knowledgeable kebele participants, farmers, key informants, community elders and leaders, religious leaders, inhabitants of forest patches, woodworkers, apiculturists and potters. During FGDs, each kebele resulted in five to seven participants representing 88 informants in 13 study sites. A total of 62 males and 26 females were involved with different age groups, genders and respective numbers of participants (20–35 years old with 12 males and five females, 36–59 years old with 24 males and 12 females and ≥ 60 years old with 26 males and 12 females). The total number of informants involved in this ethnoveterinary medicinal plant survey was 387, comprising 255 (65.89%) males and 132 (34.11%) females, and their ages ranged from 20 to 90 years. Of these, general informants were 296 (76.49%) with 179 (60.47%) males and 117 (39.53%) females; key informants were 91 with 76 (23.51%) males and 15 (16.48%) females.

They reported information on the diversity of ethnoveterinary herbal plants, their usage, threats to indigenous plant species and methods of conservation and management. In addition, participants received information about the use of medicinal plants and were involved in the collection of specimens. Each discussion was guided by the kebele administrator, an environmental protection expert, and an officer of forest and climate change, who served as local language translator for other team members during discussions. In the meetings, verbatim information was chaired and recorded by the first author (researcher). Local names of ethnoveterinary plants, habits, parts used, locations, flowering or blooming periods, time of plant part collection for remedy preparation, dosage, preparations using different methods, causes of health problems and symptoms shown by the livestock if they consume poisonous plant parts, antidotes and other important notes were discussed and recorded.

### Informant sampling techniques in the study sites

Respondents were sampled based on information from the reconnaissance survey, the FGD session at the District level, community recommendations and researcher’s observations during the initial direct interactions with informants. General informants (sampled using systematic random sampling approaches) and key/knowledgeable informants using purposive selection approaches were used for identification of traditional veterinary herbal medicine end users and practitioners following standard methods described in the literature [18, 19]. Selection of key informants relied more on information provided by recommendations of knowledgeable inhabitants, elderly people, community members and kebele administrators. General informants were sampled from the total households. The total household number (11, 908) was obtained from Soro District Finance office, Planning and Economic Development Office. This number was multiplied by the sample size then divided by total households found in each kebele. For instance, the total number of households for Bure kebele was 660 and the number of general informants was calculated as $\left(\frac{660}{11908}*387\right)$ = 21. Similar calculations were made for all kebeles and different number of informants were obtained which added up to an overall total sample size of 387 (Table 1) for the study area following this sample size determination formula [20].

**Table 1:** Sampled administrative kebeles with informants interviewed, altitudinal ranges, agroclimatic zone, and socio-demographic profile

No	Subdistrict (kebele)	GPS-altitude (m.a.s.l)	Agroclimatic zone	Total number of informants	Socio-demographic profile			
					Gender		Ethnicity	Language
					M	F	Had, Amh, Kam, Oro, Wol, Tig	Hadiyissa, Afan Oromo, Amharic
1	Shonkola	2451–2754	D	45	30	15	Had	Hadiyissa
2	Kosha	2334–2436	D	37	28	9	Had, 1 Amh	Hadiyissa, Amharic
3	Beinera	2162–2446	D	25	17	8	Had, 1 Kam	Hadiyissa, Kambatissa
4	Bambo	2082–2105	WD	17	12	5	Had	Hadiyissa
5	Wosheba	2110–2120	WD	33	19	14	Had	Hadiyissa
6	Bure	2070–2080	WD	21	14	7	Had	Hadiyissa
7	Sundusa	2042–2067	WD	44	30	14	Had	Hadiyissa
8	Share	1900–2009	WD	39	23	16	Had	Hadiyissa
9	2nd-Hankota	1975–2287	WD	33	20	13	Had	Hadiyissa
10	2nd-Oda	1758–2015	WD	26	21	5	Had	Hadiyissa
11	Ambe-lenge	1588–1665	K	21	11	10	Had, 1 Oro	Hadiyissa, Afan Oromo
12	Gebebe-lenge	1555–1565	K	25	18	7	Had, 1 Wol	Hadiyissa, Wolyitegna
13	Burye-lenge	1472–1555	K	21	12	9	Had, 2 Tig	Hadiyissa, Tigrigna, and Amharic
Total		–	13	387	255	132	381Had, 6 others	381 Hadiyissa, 6 others

*D* Dega (highland); *WD* Woinadega (temperate zone); *K* Kola (lowland); *Had* Hadiya/Hadiyissa; *Amh* Amhara/Amharic/Amharigna; *Oro* Oromo/Afan Oromo; *Kam* Kambeta/Kambatissa; *Wol* Wolyita and wolyitegna; *Tig* Tigre and tigrigna

### Data collection

Ethnomedicinal data on ethnoveterinary plants were collected using field observations, guided field walks, semi-structured interviews and focus group discussions following methods described in relevant sources [18, 21]. The semi-structured interview questions were prepared in the English language and then orally translated into Hadiyissa, informants’ mother tongue. Informant interviews were conducted individually to obtain sufficient information on livestock medicinal plant species, parts used, preparation methods, commonly treated ailments, routes of administration and dose determination. Data regarding diversity, habitat distribution, and threats to ethnoveterinary medicinal plant species were gathered from the community. Voucher specimens of all reported medicinal plants were collected from various locations in the three agroclimatic habitats by interviewing traditional medicine practitioners/healers working as key informants and general informants as well.

Important georeferenced data using the geographic positioning system (GPS), vernacular plant names, habitats and habits of each plant specimen were recorded. Voucher plant specimens were numbered and coded, pressed, dried and identified with the help of the Flora of Ethiopia and Eritrea [22–24]. The identification was verified by comparison with authenticated plant specimens found at the National Herbarium, Addis Ababa University, confirmed by taxonomic experts and finally deposited there (AAU).

### Data analysis

Microsoft Excel spread sheet software version 2016, SPSS version 25 and one-way ANOVA and values of F-tests were employed for the analysis of data on ethnoveterinary medicinal plants with the use of indigenous knowledge in various informant groups. The collected herbal ethnoveterinary data sets were analysed mainly by qualitative as well as quantitative approaches and descriptive statistics [18]. Preliminary informants’ demographic information, livestock ailments categories and ethnoveterinary medicinal plant frequency and percentages based on general features (such as forms of plant life cycle, parts used in remedy preparation, route of administration, preparation forms, means of applications and dose determination) and analysed using tables, figures and descriptive texts.

Ethnoveterinary ailments were collected from the District, and informants’ interviews were categorized to reflect on understanding of the local and indigenous uses of traditional herbal medicine and ailment signs and symptoms based on the ICPC (International Classification of Primary Healthcare) as stated by Staub et al.[25].

As a consequence, ethnobotanical scoring and ranking using values of informant consensus factor (ICF), index of fidelity level (FL), preference ranking (PR) and direct matrix ranking (DMR) were conducted for crosschecking and verification of the potential priority ethnoveterinary medicinal plant species to heal different ailments and to ensure the level of consistency as recommendations of [26–28], as well as to identify the priority species for conservation, and statistical analysis was used to create charts and graphs.

Informant consensus factor was used to describe the agreement between informants when choosing the most cited medicinal plant species that was used to treat a group of ailments in the ailment category. It was used to evaluate and to prioritize the reliability of medicinal plant data. The formula was ${\text{ICF}}=\frac{{\text{Nur}}-{\text{Nt}}}{{\text{Nur}}-1}$, where, ICF is the informant consensus factor, Nur is the number of each selected medicinal plant species use citation, and Nt is the number of selected plant species used [29, 30].

Index of fidelity level (FL = Ip/Iu × 100) was used to estimate the relative curing/healing efficacy of each potential medicinal plant species based on the proportion of respondents who agreed on its use against a given category of the ailments [19, 21, 27], where Ip is the number of informants who independently cited the importance of a species for a particular main ailment and Iu—the total number of informants who reported the same plant for any ailment [26]. In the ethnobotanical studies index of FL, it was recommended to use medicinal plants for their future phytochemical analysis, activities of antimicrobial test, characterization, bioactive chemical isolation, for drug formulation and characterization [31].

Preference ranking is defined as arranging a rank of most preferential medicinal plants that was scored for treatment of specific ailment by respondents responses following relevant sources [18, 19]. Mainly key informants were used to assess the degree of preferences of medicinal plants that were scored by informants.

DMR was used to compare multipurpose medicinal plants commonly reported for diverse use and diversity of a specific plant species using key informants following the methods [18, 19, 21]. The uses of multipurpose medicinal plants were selected from the total of confirmed livestock medicinal plants. Samples of key informants were listed and discussed the uses of the plant species. They were asked to assign and order the use values to each species (best = 5, very good = 4, good = 3, less used = 2, least used = 1 and not used = 0). The values of the average scores were given to individual medicinal plant species that were summed up and ranked. In addition, randomly selected ten (10) key informants were involved in a priority ranking exercise that focussed on perceived threatening factors of the five medicinal plant species. Direct matrix ranking score of randomly taken 10 key participants for five ethnomedicinal livestock plants for different use categories. In general, these overall ranking exercises help to check targeted indigenous plants with associated local knowledge for those claimed multipurpose indigenous plant species in the study community.

## Results

### Demographic features and indigenous knowledge on informants

Most informants belonged to the protestant (80.88%) and Hadiya ethnic groups, followed by adventists (7.75%). The occupations of most respondents were farmers (73.64%), followed by housewives (23%), and others (Table 2). Key informants reported more number of ethnoveterinary medicinal plants, and they have relatively more knowledge of their uses than general informants; they reported one or more medicinal plant species for the healing purposes of various livestock ailments. They were categorized in between young ages (20–35), adult ages (36–59) and old ages (> or equal to 60 years old) and accounted for 24.29% (94, 51 males and 43 females), 50.90% (197, 127 males and 70 females) and 24.81% (96, 77 males and 19 females), respectively. Most of the informants, 62.53% (242, 176 males and 66 females), were literate people who are able to read and write (R&W), followed by illiterate people (who cannot R and W), and 37.47% (145, 79 males and 66 females). Statistically, males (4.59 ± 3.55) have rich veterinary drug information compared with females (3.29 ± 1.37), and the difference was statistically significant *P* value (*P* < 0.05). Literates, 242 (4.43 ± 3.16) reported more average numbers of medicinal plants in the community for various ailments and this is highly significant (*P* < 0.05) than illiterates. This could be related to the fact that literates keep written information rather than oral retention of information alone. The same trend was observed considering 145 (4.12 ± 2.27) as well as distantly (4.34 ± 3.06) than nearby (4.20 ± 2) to the town. Similarly, significantly (*P* < 0.05) more medicinal plants were reported by key informants (91, 8.22 ± 4.70) than general informants 296 (2.46 ± 1.17). However, higher average number of medicinal plants were reported by elderly informants (5 ± 3.44) who are older/senior members of the community than adults (4.22 ± 2.70) and the young age (3.77 ± 2.47), though the difference was statistically non-significant (*P* > 0.05, *P* = 0.257) that informed and reported higher numbers of medicinal plants than young and adult ages (Table 3). Also, there was no significant variation that was observed among three agroecology of the District (*P* > 0.05, *P* = 0.112).

**Table 2:** Informants demographic background characteristics

	Religions	N	Per cent (%)
Religion background	Protestant	313	80.88
	Adventist	30	7.75
	Catholic	16	4.13
	Apostles	16	4.13
	Orthodox	11	2.84
	Muslim	1	0.26
Informants’ occupations	Farmers	285	73.64
	Housewives	89	23
	Unemployed	5	1.29
	Government employees	4	1.03
	Artists, artecraft, and wogesha	3	0.77
	Retired and traders	1	0.26

*N* Number of informants

**Table 3:** Statistical test of significance using one-way ANOVA on the average number of ethnoveterinary medicinal plants reported among various variables on the data collected from Soro District

Participants	Informants group	N	Average ± SD	*F* value	*P* value
Gender	Males	255	4.59 ± 3.55	13.62	0.000*
	Females	132	3.29 ± 1.37		
Age category	Young (20–35 years old)	94	3.77 ± 2.47	1.36	0.257**
	Adult (36–59 years old)	197	4.22 ± 2.70		
	Elderly (≥ 60)	96	5 ± 3.44		
Educational status	Illiterate	145	4.12 ± 2.27	3.88	0.050*
	Literate	242	4.43 ± 3.16		
Proximity to the main town	Less than 5 km	86	4.20 ± 2	4.06	0.045*
	Greater or equal to 5 km	301	4.34 ± 3.06		
Informants’ category	Key informants	91	8.22 ± 4.70	352.32	0.000*
	General informants	296	2.46 ± 1.17		
Agroclimatic zone	Dega	107	3.19 ± 3.63	2.20	0.112
	Woinadega	213	2.98 ± 4.77		
	Kola	67	1.84 ± 4.33		

*Significant difference (*P* < 0.05) at 95% conference interval for mean between groups

** non-significant (*p>0.05*), *t* (0.05, two tailed)

df = *N* − 1; 386, *N* = number of respondents = 387

### Taxonomic diversity of livestock medicinal plants (LsMPs) in Soro District

**Fig. 1:**
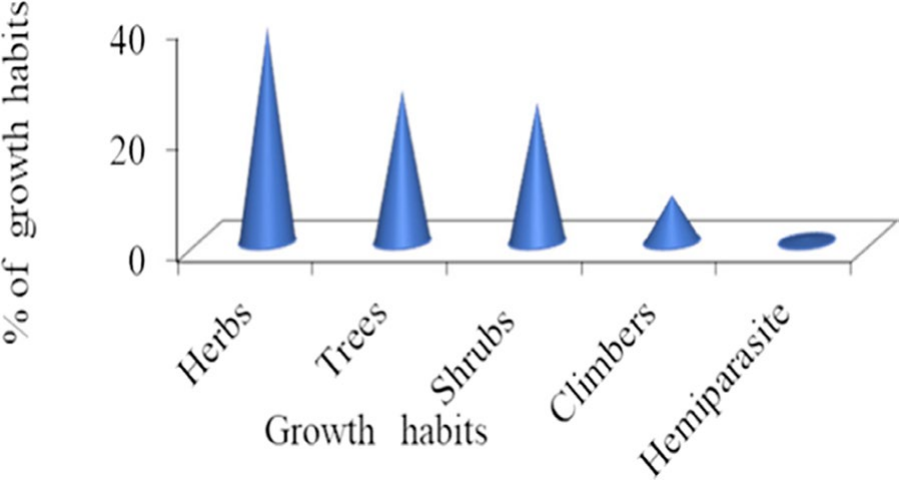
Growth habits of livestock medicinal plants in Soro District

**Fig. 2:**
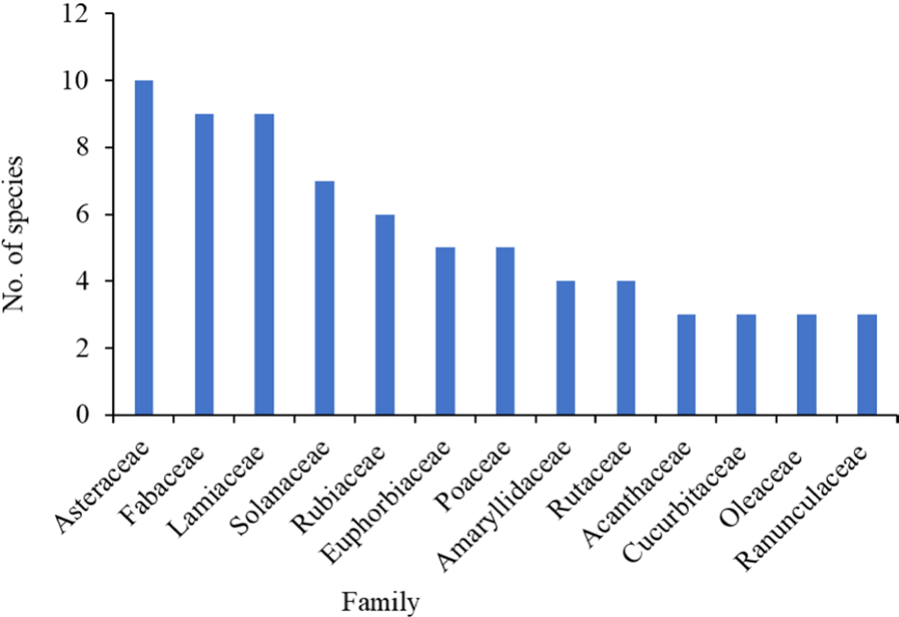
Families of livestock medicinal plants in Soro District

A total of 132 ethnoveterinary plant species belonging to 120 genera and 61 families were collected from the altitudinal ranges of 1472–2754 m.a.s.l., identified and documented. These plants are used for the treatment of different ailments of domestic livestock in the study District as reported by informants. Of these, 13 (9.85%) species are endemic medicinal plants (*). The majority about 129 (97.73%) of LsMPs are flowering plants, and there are three (2.27%) gymnosperms and 26 exotic plants. The recorded plants include 14 spices, four cereal crops, two pulses and two stimulants involved in medicinal preparations. These plants are used by the community primarily as first-aid materials to handle various health problems in livestock. Based on the results of the growth form analysis of livestock medicinal plant species, herbs contributed the highest species proportion of 51 (38.64%), followed by trees at 36 (27.27%); hemiparasites accounted for the least proportion of one (0.76%); and others lie in between (Fig. 1). These ethnoveterinary medicinal plant species have dominant families, with the highest number of plant species (Fig. 2) accounting for a different number of families and genera. Of these Asteraceae accounted 10 (7.58%) species, Fabaceae nine (6.82%) species and both with eight (6.67%) genera, and Lamiaceae nine (6.82%) species and Solanaceae seven (5.30%) species both with seven (5.83%) genera were the dominant families followed by Rubiaceae six (4.55%) species and genera (5%), both Euphorbiaceae and Poaceae five  (3.79% each) species with respective four (3.33%) and five (4.17%) genera, Rutaceae and Amaryllidaceae four (3.03%) species and three (2.50%) species with four (3.33% each), respectively; Cucurbitaceae, Acanthaceae, Oleaceae and Ranunculaceae three (2.27% each) species; three (2.50%) genera of the former family and two (1.67% each) genera of the later three families, and other 48 families were 61 (46.21%) species and 59 (49.17%) genera were also reported frequently to use for local healthcare systems of livestock. All these collected livestock medicinal plants were distributed in different living habitats, mainly wild habitats (105 species, 79.54%), some gathered from cultivated lands (27, 20.45%); of these 106 (80.30%) native species (with one  asterisk, 13 endemic species, and without asterisks, 93  indigenous species) and  26 (19.70%) introduced plant species (asterisks **) Table 4 Table 9 These LsMPs (livestock medicinal plants) were also collected in different specific localities (*i.e*., forest patches, homegardens, markets, roadsides, agricultural lands with croplands, grazing or/and grasslands). These ethnoveterinary medicinal plants were used to treat livestock ailments in the District.

**Table 4:** Values of informant consensus factor of ethnoveterinary medicinal plants used by communities of Soro District for treating certain livestock aliments

No	Ailment category	No of plants	% all plants	Use citations	% all use citations	ICF values: Nur-Nt /Nur-1
1	Lumpy Skin Disease/Ailment, FMD, blackleg, PPR, bat urine ailment, skin wound	56	42.42	198	38.08	0.72
2	Diarrhoea, abdominal pain, acidiosis/bloat, anthrax, actinobacillosis (wooden tongue), telleriosis	46	35.85	157	30.19	0.71
3	*Aspiration pneumonia*, coughing, parasitic leech, asthma	27	20.45	89	17.11	0.70
4	Livestock ascariasis (cysticercus), Babesiosis (liver ailment)	11	6.11	30	5.77	0.65
5	Coccidiasis, New Castle Disease/ Ailment	4	5.34	9	1.73	0.62
6	Eye pain (conjunctivitis)	7	5.30	14	2.7	0.54
7	Rabies, listeriosis	9	3.79	18	3.46	0.53
8	Evil eye, evil spirit, michi	3	2.27	5	0.96	0.50

### Medicinal plant parts, medicinal additives, use conditions and administration routes

The result of this study indicated that many medicinal plant parts were picked from mother plants and prepared to treat diverse types of specific livestock ailments, either in the form of single or more varied plant parts, with the use of other additives 199 (48.54%) and without the addition of additives 211 (51.46%). Ethnoveterinary medicines were prepared more with a mixture of two different plant parts, about 47 (33.33%), and the combination of more than two plant parts accounted for about 68.08% [*i.e.*, with the addition of three different plant parts 23 (16.31%), four plants 10 (7.09%), five plants eight (5.67%), six plants four (2.67%), seven plants three (2.13%) and nine plants one (0.71%)] than remedy preparation from a single plant part accounted for 45 (31.92%) preparations.

In the study area, Soro District, different informants frequently reported that various types of additives were used for the preparation of ethnoveterinary herbal drugs, and they were also used for medicinal purposes such as cold and warm water 190 (71.97%) out of 264, saliva during chewing medicinal plants 20 (7.58%), NaCl salt 16 (6.06%), milk ('Irigo') and its products 10 (3.79%), beverage/arekie five (1.89%), salty soil/locally 'Borra'*-*Hadiyissa name for salty soil occasionally given to livestock five (1.89%), cattle dry dung four (1.52%), enset ('hamicho','bu'o', 'kocho') four (1.52%) and plant latex three (1.14%). Whereas among the total number of all the different additives reported, other less frequently used additives include charcoal two (0.76%); soil from a depth of 50 cm; dry faeces of donkeys one (0.38%); and others one (0.38% each) like petroleum gas, penicillin and sprite were used by local people to mix with medicinal plants.

Some of those additives were used as antidotes for various traditional drug problems; these include the use of excessive water, milk (’Irigo’) and its products. Of all, water is the universal natural mixing medium among different dilution solvents and serves as a universal additive. Certain plants, such as *Carduus schimperi* and *Clutia abyssinica,* have medicinal uses in cases where toxic or poisonous plants are eaten by livestock to neutralize their bad effects. Key informants reported that they also have nutritional uses for livestock fattening.

**Fig. 3:**
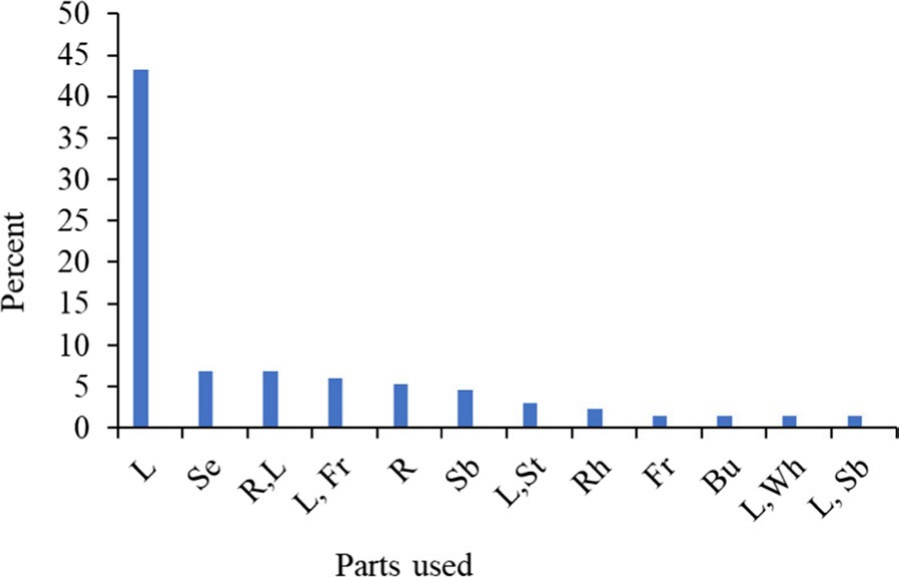
Parts of livestock medicinal plants used in Soro District. *Note*: Key: L = leaf; Se = seed; R, L = root or leaf; L, Fr = leaf or fruit; Sb = stem bark; L, St = leaf or stem; Rh = rhizome; L, Wh = leaf or whole part; Bu = bulb; L, Sb = leaf or stem bark and No = number

The data collected showed that fresh leaves (57 species, 43.18%) were most frequently used in familiar preparations, followed by seeds (6.82%), roots with leaves (6.82%), fruits with leaves (6.06%), root alone (5.30%), stem bark alone (4.55%), leaf with stem (3.03%), Rh (2.27%), Fr, Bu, L with Wh, L with Sb parts two (1.52% each) and with other parts (Fig. 3). Moreover, many other parts with one (0.76% each) were also used by healers, including flowers and inflorescences, whole parts, leaves and resin.

**Fig. 4:**
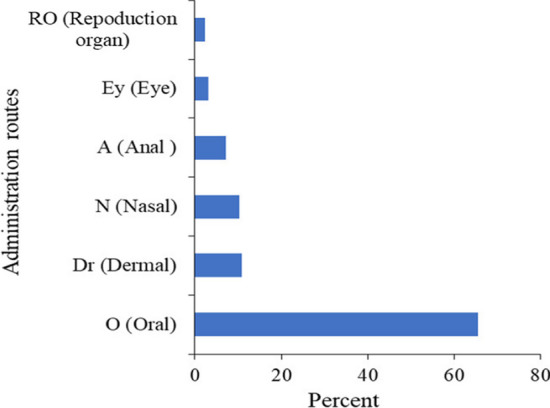
Reported routes of administration of livestock medicinal plants medicines. *Note*: % = per cent; no = number

The various traditional herbal medicine parts were used in fresh form about 109 (82.58%), followed by dry, 12 (9.09%) and 11 (8.33%) fresh/dry parts. The results of the reported analysis of the application route of this study pointed out the relative numbers of varied routes of administration for traditional medicines to treat different types of livestock ailments. Oral administration through the mouth was the most commonly used route 191 (65.41%), followed by dermal 32 (10.96%), nasal 30 (10.27%), anal 21 (7.19%) and others like optical and reproductive organ, whereas both the ear and spraying plant extract prepared by herbal medicine on the physical (external) environment to against or kill ailment-causing agents similarly contributed the least number one (0.34%) each of the 292 total reports (Fig. 4).

### Forms of medicinal preparation and application methods

**Fig. 5:**
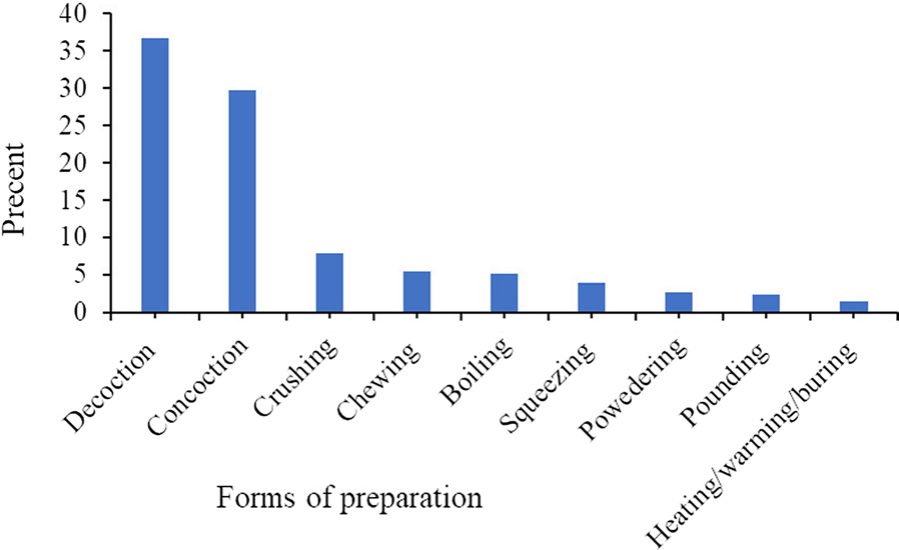
Forms of medicinal preparation

Results of the analysis of medicinal preparation of medicinal plants showed that decoction 94 (36.7%) for remedy preparation from a single medicinal plant species made the largest proportion, whereas concoction 76 (29.7%) by mixing plant material from different species came in the next place following by crushing 20 (7.8%), chewing 14 (5.5%), boiling 13 (5.1%) and others (Fig. 5). In addition, some of the herbal preparation out of the 256 total preparations, pasting and infusion accounted three (1.2% each); burning two (0.8%); with others cooking/roasting, holding, chopping and without processing accounted one (0.39% each).

**Fig. 6:**
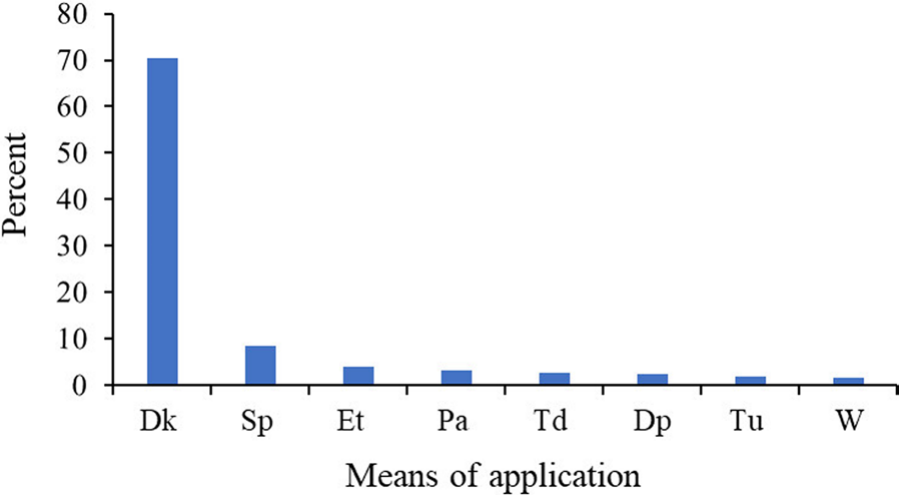
Means of livestock medicinal application. *Note*: Key: Dk = drinking through the mouth; Sp = spitting through the nose; Et = eating; Pa = pasting to the painful area; Td = tying on the painful area; Dp = dropping to the eye; Tu = touching the external painful area; W = washing the body; and % = a symbol of percent

Ailment treatment through drinking by mouth (181 (70.43%) of the 257 total reports) was widely used, and the most common method for traditional remedies application method following spitting through the nose (8.56%) and eating (3.89%) came up in higher proportions flowing pasting, others (Fig. 6). Whereas inhaling the steam by nose, smearing (creaming/ontiment) to the body and smoking through nose accounted three (1.17% each); swallowing through mouth two (0.78%); and spraying to the physical environment, sprinkling on the wound and inserting to the body each one (0.39%).

### Marketability of livestock medicinal plants

Marketable traditional medicinal plants, both medicinal and species, were recorded in four sampled and surveyed markets from three agroecological areas (Gimbichu (Dega), Jajura (Woinadega), Kosha (Dega) and Humaro (Kola) local markets). They were mainly purchased for medicinal use and accounted for seven plant species (5.30%). These medicinal plants were *Ajuga integrifolia (***Annaamura**-Hadiyissa/Had.), *Antherica* sp (**Dashshi maracca**-Had.), *Asparagus africanus* (**Hundufaanna**-Had.), *Artemisia absinthium (*Naatira-Had.) sold for sources of spices and medicinal uses, *Nicotiana tabacum* (**Tambaa'i-koshsho'o**-Had.), *Securidaca longepedunculata* (**Mukke'e**-Had.), *Hagenia abyssinica* (**Suuxo**-Had.), were recorded from the three agroclimatic open markets, and they were sold and purchased for the purpose of traditional medicine.

The prices of each species varied from market to market. For example, the prices of a bunch of **Dashshi maracca**-Had (*Antherica* sp)., and **Mukke’e**-Had (*S. longepedunculata*), and a mug/water glass of **Suuxo**-Had (*H. abyssinica*) were sold and purchased each 40–50 EthBirr. One coffee or tea cup of Naatira-Had. (*A, absinthium*), **Tambaa’i koshsho'o**-Had. (*N. tabacum*, and were sold by 10–20 EthBirr.

As reported for decades in the study area, some medicinal plants were commonly sold for the purposes of livestock herbal medicines, such as *Ajuga integrifolia* (**Annaamura**-Had.), *Echinops kebericho* (**Toosa**-Had.), and *Hagenia abyssinica* (**Suuxo**-Had.), were sold and purchased in excess for functions of traditional medicine. However, nowadays, due to various impacts, these plant species have become locally extinct in the area because of human activities such as harvesting for various uses and the removal of those potential plants from the community. Thus, they require systematic *in situ* and *ex situ* conversation plans to conserve them with the relationships of people living there.

### Ethnobotany of the best livestock plant species in Soro District

In the study area, Soro District, the highest ICF values were recorded for a group of ailments under dermatological ailments (0.72) followed by gastro-intestinal (0.71) and respiratory (0.70) ailments which depicted the agreement on knowledge of medicinal plants used to treat best by the community (Tables 4 and 5).

**Table 5:** FL values of 20 most frequently used ethnoveterinary medicinal plant species of Soro District

No	Livestock ailment	Ethnoveterinary medicinal plant	I_p_	I_u_	FL values (%)
1	Rabies	*Datura stramonium*	9	9	100
2	PPR (Pestedes petits ruminants)	*Dodonaea viscosa* subsp*. angustifolia*	17	17	100
3	Evil eye (sprit)	*Asparagus africanus*	25	25	100
4	Foot and Mouth Disease (FMD)	*Croton macrostachyus*	49	50	98
5	*Aspiration pneumonia*	*Albizia schimperiana*	56	58	96.55
6	Diarrhoea	*Brugmansia suaveolens*	70	76	92.10
7	Retained placenta	*Cyphostemma pannosum*	19	21	90
8	Blackleg	*Cyathula uncinulata*	9	10	90
9	Anthrax	*Bersama abyssinica*	60	67	89.55
10	Acidiosis (bloat)	*Scepocarpus* *hypselodendron*	40	45	88.89
11	Livestock *ascariasis*	*Coleus abyssinicus*	20	23	86.96
12	Bat urine ailment /jaundice	*Momordica foetida*	39	48	81.25
13	Toxicity curing	*Clutia abyssinica*	26	32	81.25
14	Livestock mites, fleas and lice	*Calpurnia aurea*	14	18	77.78
15	Parasitic leech, snake bite, insects	*Nicotiana tabacum*	28	40	70
16	Trauma (broken bones); placental	*Ensete ventricosum* (red)	15	22	68.12
17	Snake bite (injection of venom)	*Sida rhombifolia*	8	13	61.54
18	Dingetegna	*Euclea divinorum*	16	30	53.33
19	Asthma/stenosis, livestock tumour	*Euphorbia abyssinica*	15	29	51.72
20	Livestock hepatitis /jaundice	*Clematis hirsuta*	6	12	50

Simple preference ranking exercises/practice with the best ten (10) randomly chosen key knowledgeable informants for the most 10 livestock plants were reported against the most prevalent ailment category among gasto-intestinal ailments in the study sites. It is effectively used for treating LSD which was repeatedly reported in the study District. Samples of key informants were involved in the interview and asked to assign the number one for the least effective medicinal plant species and 10 for the most effective plant. As a result, *C. macrostachyus* was ranked first and *X. americana* ranked second, *A. sativum* was ranked third, and *J. procera* was ranked fourth; however, *Z. officinale* was ranked in the lowest categories (Table 6).

**Table 6:** Ranking values of ten most preferred medicinal plant species widely used to treat the dermatological ailment known as Lumpy Skin Disease (LSD)

Ethnoveterinary plant species used to treat Lumpy Skin Disease	Randomly taken key informants A-J										Total score	Rank
	A	B	C	D	E	F	G	H	I	J		
*Croton macrostachyus*	10	8	10	9	7	9	8	7	10	8	86	1st
*Ximenia americana*	10	9	7	8	9	7	10	5	1	9	75	2nd
*Allium sativum*	6	5	6	6	8	8	9	7	9	10	74	3rd
*Juniperus procera*	1	7	5	10	6	5	8	9	4	8	63	4th
*Nicotiana tabacum*	10	5	4	1	5	9	9	3	5	7	58	5th
*Euclea divinorum*	8	7	5	6	4	6	5	4	6	1	52	6th
*Momordica foetida*	7	6	8	4	6	5	6	3	1	5	51	7th
*Solanum incanum*	5	1	6	1	7	3	5	1	4	5	38	8th
*Ocimum spicatum*	3	4	1	3	1	2	1	4	5	3	27	9th
*Zingiber officinale*	2	1	3	2	5	1	1	3	1	4	23	10th

*Key information:* Total scores in the table indicated the ranks which assigned to ethnoveterinary medicine rely on their efficacy with highest number 10 and the least one to treat a given ailment

### Multi-use multipurpose traditional livestock medicinal plant species and conservation activity

The result of the average output of the direct matrix ranking score of 10 key informants for five use diversities showed that some multipurpose ethnoveterinary species are highly exploited for firewood, charcoal and house construction and utensils rather than the use of medicinal values. These 1st, 2nd and 3rd ranked plant species became locally extinct and endangered due to the relatively highest harvesting activities of each plant species for the sake of various functions (Table 7). Thus, these medicinal plants were used for livestock ailments and they needed conservation priority based on the present status in the communities of the study area.

**Table 7:** DMR scores of five ethnomedicinal plants used to treat livestock ailments by ten key informants

Ethnoveterinary medicinal plant species	M	Fo	Con &Ut	Sha	Ch	Fw	Total score	Rank
*Olea welwitschii*	1	3	5	0	5	5	19	4th
*Afrocarpus falcatus*	3	0	5	5	3	4	20	3rd
*Combretum molle*	3	2	5	2	5	5	22	2nd
*Apodytes dimidiata*	3	0	1	1	4	4	13	5th
*Prunus africana*	5	1	5	3	5	5	24	1st
Total score	15	6	21	11	22	23	98	
Rank	4th	6th	3rd	5th	2nd	1st		

*M* Medicine, *Fo* Fodder, *Con & Ut* Construction and utensils, *Sha* Shade, *Fw* Firewood, *Ch* Charcoal

Here, five is given for the highest score number, and the least score is given by one. Agricultural expansion, new settlements, local charcoal and overgrazing were the main threats to ethnoveterinary medicinal plants. For this reason, educational training with economic support is a prominent need for relatively well-known intellectual and knowledgeable herbal medicine practitioners in the District to use herbal medicine in a sustainable way.

### Livestock aliment categories, types and methods of ailment diagnosis

A total of fifty (50) livestock ailments were identified and categorized from the reports of different stakeholders (Table 8) including local name and ailment categories. In this study, the most commonly and repeatedly reported livestock ailments were recorded and identified. The occurrence of these identified veterinary ailments was classified with the help of veterinary experts from the District. They were collected from the District FGD report, the District Veterinary Health Office report and different study sites as follows: FMD, Lumpy Skin Disease/Ailment (LSD/A), actinobacillosis (wooden tongue), anthrax, babesiosis, acidiosis (bloat), blackleg, livestock trypanosomiasis, New Castle Disease/Ailment (NCD/A), PPR (Peste des petits ruminants-ailment of goats) and diarrhoea were the top 10 frequently reported livestock ailments, following coccidiasis, *aspiration pneumonia*, abdominal pain, rabies, eye pain, evil eye/evil spirit and livestock ascariasis. They frequently attack bovines, equines, sheep, goats and poultry are treated with indigenous herbal medicines by local people, who said they mainly rely on different medicinal plant species to treat them in addition to modern healthcare services.

**Table 8:** Livestock ailments recorded in the study area (Soro District)

No	Identified livestock aliments	Vernacular name (Hadiyissa/Had.)/Few in Amharic (Amh.)	Affected Livestock Type	Ailment Category
1	Foot and Mouth Disease/Ailment, FMD/FMA (it removes hooves from cattle)	Anjichcho/nidifa	Infects bovines (cattle)	Dermatological inflammation
2	Lumpy Skin Disease/Ailment (LSD/A)	Loophphi jabbo, ye qoda gurubiribita (Amh.)	Infects bovines	
3	Blackleg (inflammation of skeletal and cardiac muscles, severe toxemia and high mortality)	Moggolle’i/hafachchi jabbo	Infects bovines	
4	PPR (Peste des petits ruminants- ailment of goats which creates wound infection around the mouth).; shoat pox	Fanxaaxa/kurkusso’o	Mostly attacks both sheep and goats	
5	Epizootic lymphangitis	Gammaam tusha	Equines (large numbers of livestock of horses, donkeys and mules)	
6	Bat urine ailment (cause skin ulceration)	Cii’i xisso, yewof beshita (Amh.)	Attacks all livestock	
7	Mastitis (breast-dermatologicao)	Anuu’n jabbo, ye desta bashita (Amh.)	Bovines	
8	Actinomycosis	Korossa/gergeeda	Bovines	
9	Skin wound (freshly skin cutting)	Xiige’oo mada/omara	Attacks all livestock	
10	Sore-with pus	Maraam omara	Attacks all livestock	
11	Skin ailment	Omachchi jabbo	Attacks all livestock	
12	Back sore	Afa’l gambaxa	Attacks equines: horses, donkeys and mules	
13	Livestock tumour (venign tumour/external tumour)	La’l xeenxira	Infects bovines, equines	
14	Swelling body	Orachchi dashshimma	Bovines	
15	Anthrax	Xiinxichcho, abassanga (Amh.)	Infects bovines, equines, sheep	Gastro-intestinal
16	Livestock trypanomiasis (due to bite of tsetse fly)	Sute’e, gandi (Amh.)	Infects both bovines and equines	
17	Coccidiosis	Xiiga edaakkoo aadora	Infects poultry	
18	*Fowl typhoid*	Cii’i soko’i gaayyo’o	Infects poultry/cholera	
19	Livestock hepatitis/jaundice (liver ailment cause yellow skin (Ectruse)	Afa’l jabo	Attacks all livestock	
20	Diarrhoea	Alooyya te’im godaphphi aadite	Attacks all livestock	
21	NCD/A (New Castle Disease/Ailment)	Kembesha, feangil (Amh.)	Poultry ailment	
22	Acidiosis (bloat)	Godabduubimma/quruuro’o	Attacks all livestock	
23	Faciolosis	Mure’e, loomme/ wocwoca	Bovines, sheep and goats	
24	Actinobacillosis (wooden tongue)	Soorgassa/allabo mooradisoo jabbo	Bovines	
25	Telleriosis/Anaplasmiosis (spleen enlargement)-tick-borne ailment)	Hilleeffi jabbo/suruulli-jabbo	Bovines and sheep	
26	Toxicity (poisonous)	Marzi yoo mutaano itimmi jabbo	Poisonous bovines, sheep and goats	
27	Abdominal pain/abdominal ache	Bashsha’na	Bovines and sheep	
28	Constipation	Shilli gokka	Bovines, sheep, goats	
29	Nasal bote (parasitic leech)	Urulla	Attacks all livestock	Respiratory
30	Asthma (stenosis of respiratory organ)	Saallaaqa/shiinqa	Equines	
31	Pasteurellosis (livestock TB)	Suqqo’i/qadafa’l jabbo	Attacks bovines	
32	*Aspiration pneumonia*	Siniqa	Bovines, sheep and goats	
33	Insects’ infection	Sa’n kochcha’na	Bovines, sheep, goats	
34	Livestock mites, flea and lices (mange mite/otitis)	Baalqaanca, ibiiba, cibe’e	Attacks all livestock	Ecto-parasitic
35	Babesiosis (liver ailment)	Waadamuuncho	Attacks all livestock	Endo-parasitic) (protozoan)
36	Cysticercus (bovis, ovis/livestock ascariasis)	Hensheeshsha	Bovines, goat, sheep	Endo-parasitic
37	Rabies (dogs, cats, foxes, wolves, insectivorous and fruit-eating bats)	Machchaaru wish jabbo	Attacks all livestock	Neurologic
38	Snake bite	Hamash qasimma	Attacks all livestock	
39	Listeriosis	Horoore jawwaa’aa do’isimma, azurit (Amh.)	Attacks all livestock	
40	Tetanus	Teetaanoosa	Attacks all livestock	
41	Conjunctivitis (eye pain)	Illi xisso	Attacks all livestock	Orbital
42	African horse sickness (para-orbital eye lob enlargement/inflammation (AHS)	Ille fugoo jabbo	Infects equines	
43	Trauma (injury, fracture, broken bones, muscles deformities, blood accumulation in the body)	Aphphixximma/madimma	All forcefully heated livestock that cause blood accumulation	Orthopaedics
44	Arthritis (gouge or mondy-morning ailment)	Luquc/hoong jabbo	Mostly attacks equines/horses, donkeys and mules	Musculo skeletal
45	Reproduction problem	____	Bovines	Infertility, fertility or unable to fertility
46	Retained placenta (foetal placenta membrane remain)	Maqqeer gatimmi jabbo	Bovines, sheep, goats, horses and donkeys	Placental
47	Michi	Michcha	Attacks all livestock	Fibril illness
48	Dingetegn	Xokka/qasimmi jabbo	Attacks all livestock	Any aliment
49	Evil eye	Manni ille	Attacks all livestock	Other
50	Evil spirit	Goromota	Attacks all livestock	Other

Of the reported potential livestock ailments that commonly cause health problems, most of them were categorized into dermatological (including 15 types of ailments, 30%), gastrointestinal (14 types of ailments, 28%) and respiratory (five types of ailments, 10%). Dermatological ailments category is the most common category, followed by gastro-intestinal and respiratory ailments. Informants use a single uni-medicinal plant or more than one plant (a poly-medicinal plant) in the study area to treat different ailments. Other remainder ailment categories and their numbers like parasitic (ecto- and endo-parasitic) and neurologic ailment together six ailments (each three types) in the proportion of 12% (each type 6%); orthopaedics, musculoskeletal, infertility, placental and fibril illness together six ailments (each one ailment) and accounted 12% (each 2%); orbital and other ailments together four ailments (each two types) accounted 8% (4% each).

Different key and general informants reported the use of a single species to treat many different livestock ailments and the combination of two or more different medicinal plant species involving either the same or different parts in the study area to treat different specific ailment types (Table 8). For example, *Momordica foetida* was used to treat 16 (4.91%) ailments of the 326 total frequently reported livestock ailments alone or more in combination, which was used to treat bovines/cattle LSD; evil eye, evil spirt, diarrhoea, dingetegna, acidiosis (bloat) and bat urine of all livestock were treated; epizootic lymphangitis of equines (horses, donkeys and mules); anthrax of bovines, equines and sheep; telleriosis (anaplasmiosis-spleen enlargement due to tick-borne) of bovines and sheep; actinobacillosis that causes wooden tongue of bovines; abdominal pain or ache in all livestock; to treat body swelling and blackleg pain in bovines with a combination of *Hesperocyparis lusitanica* and *Euclea divinorum*; *Nicotiana tabacum* was used to treat 15 ailments (4.60%), e.g. LSD, pasteurellosis (livestock TB, *i.e*., livestock tuberculosis) or coughing, and blackleg of bovines with a combination of *Allium sativum* and other four medicinal plant species (Table 9); insect infection and *aspiration pneumonia* of bovines, sheep and goat; anaplasmiosis of bovines and sheep; back sore of equines; skin ailments, wound or sore, swelling, nasal bote, eye pain, acidiosis and snake bite (venom injection) of all livestock were treated.

**Table 9:** List of medicinal plants for treating livestock ailments in Soro District with the mode of preparations and applications

Scientific name	Family	Local name (Hadiyissa)	Ailment treated (Hadiyissa/Had. /Amharic/Amh.)	Ha	PU	CP	Medicinal plants and applications include FP (preparation form), MT (means of treatment), and RA (root of administration)	UR	VN:MH:
*Achyranthes aspera* L	Amaranthaceae	Hoffi qaccabba	*Aspiration pneumonia*	H	L	F	Crushed, squeezed, and spitted the decoction juice to the nose via nasal administration	2	MH-75
*Acmella caulirhiza* Delile	Asteraceae	Bishibisha	Pregnancy (fertility)	H	Fl	F	Crushed with leaves of *C. macrostachyus* and *O*. *rochetiana*, enclosed with fibre, and inserted in the reproduction organ for six hours after copulation in three days	2	MH-42
			Bat urine ailment (jaundice)		Fl, R, Wh	F	Crushed either part, mixed in water, and washed the body skin	1	
*Aframomum corrorima* (A. Braun) P.C.M. Jansen*	Zingiberaceae	Wokkaashsha	Abdominal pain	H	Se	D	Fresh or dry leaf or fruit of *C. sativum* and *R. chalepensis*, crushed with a bulb of *Z. officinale* and NaCl salt, homogenized in cold water, and drunk in a one-litre concoction via mouth or anus; oral or anal	14	MH-88
			Acidiasis (bloat)	H	Se	D	Seed of *N. sativa* with leaf and fruit of *C. sativum* and *R. chalepensis* crushed with bulb of *Z. officinale* and *A. sativum* with common salt or NaCl, homogenized in cold water, and drunk one-litre concoction via mouth and anus; burning or glowing with hot metal or iron in fire	6	
							Crush the leaf of *S. hypselodendron* and the leaf of *G. robusta,* mix with water, and drunk one litre	1	
							Powdered with *E. globulus* leaf and *C. macrostachyus*, mix with water, and drunk one litre	1	
			*Aspiration pneumonia*	H	Se	D	Seed is chewing with NaCl and spitting to the nose	2	
			Trauma (blood accumulation in the body)	H	Se	D	*A. corrorima* seed with *G. amygdalinum* fresh leaf crushed or fresh or dry leaf or fruit of *C. sativum and R. chalepensis*, squeezed, and drunk one litre of concoct	1	
			LSD/A	H	Se	D	Crushed with a dry leaf of *N. tabacum*, a bulb of *A. sativum*, a rhizome of *Z. officinale*, and a fresh pod of *C. frutescens*, pounded it in water and drunk a one-litre	1	
*Afrocarpus falcatus* (Thunb.) C.N. Page	Podocarpaceae	Digiba	Rabies	T	L	F	Seven buds, crushed with fresh leaves of *T. asiatica,* fresh stem bark of *S. oxyacanthum*, and *E. capensis*, are mixed with water and drunk in three coffee cups orally	7	MH-34
*Agave sisalana* Perrine. **	Asparagaceae	Aanci haqqa	Swelling	S	L	F	Boiled or heated in fire and touch on the painful part; dermal	4	MH-95
			NCD/A	S	L	F	Heated/boiled in fire and squeezed the sap, added fresh or spicy butter, and gave it to eat alone or drunk mixed with a syringe by opening the beak; drunk decoction	2	
*Ajuga integrifolia* Buch. -Ham. Ex D. Don	Lamiaceae	Annaamura	Evil eye (evil spirt)	H	L	F/D	Crushed or powdered, mixed with water, and drunk one litre or jug decoction through the mouth; oral route	1	MH-51
*Albizia schimperiana*, Oliv	Fabaceae	Maande'e	*Aspiration pneumonia*	H	Sb	F	Stem bark is chewing and spitting or sniffing at the nose	56	MH-26
*Allium cepa* L.**	Amaryllidaceae	Kashar shunkurutta	Pasteurellosis/ livestock TB	H	2Bu	F	Leaf of *C. arabica*, fresh or dry two bulbs of *A. sativum,* one rhizome of *Z. officinale*, and pods of *C. frutescens* drunk one litre of concoction given orally	2	MH-92
			Actinomycosis (Ankulicho-Had.)				Three-four bulbs *of A. sativum* crushed with one bulb of *A. cepa*, half of rhizome of *Z. officinale,* and one fruit of *C. aurantiifolia*, and drunk one litre	2	
*Allium sativum* L.**	Amaryllidaceae	Tuma	Lumpy Skin Disease/Ailment (LSD/A)	H	Bu	F/D	*N. tabacum* dry leaf, mixed with fresh *Z. officinale* rhizome, fresh leaf or root of *M. foetida*, drunk one litre for three, three, or four times in three-day intervals or until healed; burn with hot metal or heated or glowed iron in fire	42	MH-90
			Swelling	H	Bu	F/D	Fresh leaves of *C. macrostachyus* are crushed with dry leaves of *N. tabacum*; one whole bulb of *A. sativum* is mixed in water; it then stays for one to two days and drunk one-litre concoction through the mouth; oral	3	
							Ground the dry leaf of *N. tabacum* with NaCl salt together with the leaf or fruit of *R. chalepensis* and *C. sativum*, the seed of *A. corrorima*, the bulb of *A. sativum*, and *Z. officinale* mixed together with butter, and then one litre of the solution is drunk via mouth and anus	4	
							Seed of *A. corrorima* and *N. sativa* with leaf and fruit of *C. sativum and R. chalepensis* crushed with rhizome of *Z. officinale* with common salt, homogenized in cold water, and drunk one-litre concoction; burn with hot or glowing metal or iron in fire; drunk one cup petroleum	2	
							Fresh or dry bulbs are mixed with dry *N. tabacum,* pounded, and drunk one-litre concoction via mouth; oral		
			Coughing	H	Bu	F/D	Crushed with dry leaves of *N. tabacum* and *Z. officinale* rhizome, then mixed the powdered with water and drunk a litre concoction orally given		2
			New Castle Disease/Ailment (NCD/A)	H	Bu	F/D	Fresh *Z. officinale* is crushed, pounded with two to three fresh pods of *C. frutescens*, and drunk in three to four drops by syringe	2	
			*Aspiration pneumonia*	H	Bu	F/D	three to four bulbs are crushed and inhaled through the nose	1	
			Telleriosis/anaplasmiosis	H	Bu	F/D	Crushed with fresh rhizome of *Z. officinale*, mixed with arekie, and drunk one litre of concoct; oral	1	
			Constipation	H	Bu	F/D	three to six bulbs crushed with fresh/dry leaf/fruit of *C. sativum*, and drunk one-litre concoction	1	
*Aloe* sp.	Asphodelaceae	Geneeno'o	Swelling, wound	S	L	F	Boiled in fire, warmed, and touched/wrabbing the swollen area; crushed, pounded, and drunk one litre	8	MH-184
			Diarrhoea	S	L	F	Leaf is crushed, pounded, and drunk one-litre infusion	1	
*Antherica* sp.**	Anthericaceae	Dashshi maracca	Body swelling	H	Rh	F/D	Freshly crushed, pounded with water, and drunk one litre	2	MH-380
*Apodytes dimidiata* E. Mey.ex Arn	Metteniusaceae	Mewwa	Bat urine ailment	T	L	F	Crushed *U. simensis*, mixed it with water, and drunk one-litre infusion through the mouth	2	MH-253
*Artemisia absinthium* L.**	Asteraceae	Naatira	Livestock trypanomiasis	H	L	F	Leaves and buds are crushed, pounded with water, and given half a litre by oral and anal administration	2	MH-87
			*Aspiration pneumonia*	H	L	F	Chewed with fresh leaves of *C. citratus* and *R. cordifolia*, bud of *P. dodecandra*, and seed of *A. corrorima*, spit the juice to the nose	1	
*Asparagus africanus* Lam	Asparagaceae	Hundufaanna	Evil eye (evil spirit)	H	R, Wh	F/D	Its parts are crushed, pounded in water, and drunk from one water glass to one litre	20	MH-198
						F/D	Whole parts are crushed and washed with the pounded infusion in the morning	3	
			Coughing	H	R, Wh	F/D	Crushed and pounded in water, drunk one litre or glass	1	
*Balanites aegyptiaca* (L.) Delile	Zygophyllaceae	Baddanno'o	Arthritis (gouge)	T	L	F	Crushed fresh leaf, pounded, and given one litre of anal and oral administration	1	MH-16
*Bersama abyssinica* Fresen	Francoaceae	Koraqqa	Anthrax	S	L	F	Crushed, pounded in cold or warm water, and drunk one glass or mug	5	MH-80
			Swelling	S	R	F	Crushed, pasted, and tied to the painful area	1	
*Brucea antidysenterica* J.F. Mill	*Simaroubaceae*	Ciiroonta	Blackleg	S	R	F	Crushed and pounded it in water and eaten the solution	1	MH-30
*Brugmansia suaveolens* (Humb. & Bonpl. ex Willd.) Sweet. **	Solanaceae	Qadaalli fiita	Diarrhoea	S	R	F	Crushed with leaves of *E. divinorum*, drunk one-litre concoction by mouth	8	MH-201
			Acidiosis (bloat)	S			Crushed with leaves of *E. divinorum*, drunk one-litre concoction by mouth	2	
*Calpurnia aurea* (Aiton) Benth	Fabaceae	Senna	Livestock mites, flea and lices (skin ailments /mange mite)	S	L	F	Crushed, pounded with water, wash the skin by tiding mouth to treat dermatophytes; fata if it swallowed to mouth	14	MH-27
*Capsicum frutescens* L. **	Solanaceae	Mixmixo'o	Coccidiosis	H	Pd	F	Crushed mixed with penicillin; added water and mixed butter; swallowed the bolus through the beak of the hen	7	M-91
							Fesh pods with the rhizome of *Z. officinale* crushed together, mixed water, squeezed the juice, and drunk the drops by syringe through the beak	1	MH-24
			Spleen enlargement	H	Pd	F	Crushed and drunk one glass to half of litre with water	1	
			LSD	H	Pd	F	Fresh pods with dry *N. tabacum*, fresh or dry bulb of *A. sativum*, fresh rhizome of *Z. officinale*, and dry seeds of *A. corrorima* crushed, drunk one litre orally	1	
			NCD	H	Pd	F	Two–three fresh pods with two full fresh or dry bulbs of *A. sativum* and one rhizome of *Z. officinale*, crushed, and drunk by syringe through the beak or mouth	1	
			Pasteurellosis (livestock TB)	H	Pd	F	Drunk one litre with crushed or roasted *C. arabica* leaf, *A. sativum* two bulbs, *A. cepa* one bulb, and *Z. officinale* one rhizome	1	
*Carduus schimperi* Sch.Bip	Asteraceae	Hallutta	Pregnancy (fertility)	S	R	F	Crushed with fresh three buds of *C. macrostachyus* and three buds or flowers of *R. abyssinica* crushed enclosed fibre and inserted in the female sex organ by tying the lower tip of the enclosed at the tail for 30 min. to occur pregnancy	5	MH-328
							Three fresh buds of *C. macrostachyus* crushed with fresh roots or fruits of *O. rochetiana* were enclosed, pounded, or wrapped in fibre, and inserted into the female sex organ by tying the other tip on the tail for 30 min	1	
			Poisonous/toxicity by eating plants	S	R	F	Crushed, mixed with water, and drink one litre of infusion; or drink one litre of milk alone and soil immediately for eating fresh leaves of *A. salicifolia,* and germinating two–three leaves contain *S. bicolor*; cause body fatness	3	
*Carissa spinarum* L	Apocynaceae	Qoqombe'e	Swelling	S	R	F	Fresh roots were crushed, boiled, and drunk in one glass	2	MH-328
*Citrus x aurantiifolia* (Christm.) Swingle. **	Rutaceae	Loome'e	Actinomycosis	S	Fr	F	One whole fruit with three-four bulbs of *A. sativum* crushed with half a rhizome of *Z. officinale*, one bulb of *A. cepa*, and drunk one-litre concoction, oral administration	1	MH-208
*Clematis hirsuta* Perr. & Guill. *	Ranunculaceae	Hoffi fiida	Livestock hepatitis /jaundice	Cl	L	F	Crushed with fresh leaves of *C. macrostachyus* and drunk one-litre concoction via oral	2	MH-44
*Clematis longicauda* Steud. ex A. Rich. *	Ranunculaceae	Lob fiida	Babesiosis	Cl	L	F	Boiled, crushed, and mixed soil salt (‘Borra’-Had.,), and drunk one-litre infusion	4	MH-43
			Acidiosis (bloat)	Cl	R	F	Roots are crushed, pounded and drunk one litre	2	
			Pregnancy (fertility)	Cl	Bd		Crushed with buds of *C. macrostachyus* and buds of *O. rochetiana,* enclosed fibre and insert in female reproduction organ for 30 min. or occurrence of pregnancy or three days	2	
*Clutia abyssinica* Jaub. & Spach	Peraceae	Shum xiigeeshsho	Toxicity/poisonous plant eating livestock	S	L	F	Crush and drunk a one to two-litre infusion for the treatment of poisonous plants (*A. salicifolia*) eaten by livestock. Uses for livestock's fatness	4	MH-40
			*Aspiration pneumonia*	S	R, Bd	F	Crushed it, mixed with water, and drunk one litre of squeezed liquid	2	
			Actinomycosis	S	R	F	Crushed, mixed with water and drunk one litre	1	
*Coffea arabica* L	Rubiaceae	Buna	Abdominal pain, livestock TB	S	L	F	Crushed with one whole rhizome of *Z. officinale* and drunk one litre concocted	4	MH-52
			*Aspiration pneumonia*	S	Se	D	Chewed with NaCl and spited the juice to nose	1	
*Coleus abyssinicus* (Fresen.) A.J. Paton. *	Lamiaceae	Bobaanqa	Cysticercus, bovis, ovis (ascariasis), diarrhoea	S	Bd	F	Crushed, mixed with water, and drunk one mug of two litre’s; decoction of orally calf to adult	7	MH-56
*Colocasia esculenta* (L.) Schott	Araceae	Gabija	Nasal bote (parasitic leech)	H	Rh	F	Crushed the fresh leaves of *E. globulus* and drunk the concoction	1	MH-191
*Combretum molle* R. Br. ex G. Don	Combretaceae	Goonchi habulle'e	Diarrhoea	T	R, Rb	F	Crushed, powdered, and mixed in cold or warm water, and drunk a one- to two-litre decoction via oral	1	MH-193
*Commelina benghalensis* L	Commelinaceae	Lob gu’ma	Constipation	H	L	F	Crushed, mixed with water, decoct, and drunk three-litre or two-litre *Lagenaria siceraria* drinking material (Bulle’e’-Had.); oral	4	MH-81
*Cordia africana* L	Boraginaceae	Weddeeshsha	*Aspiration pneumonia*	T	L, Sb		Crushed, decocted and closed with fibre and spited to nose; nasal route	3	MH-115
			Acidiosis	T	L		Fresh leaves are crushed, mixed with water, and drunk one-litre decoction via mouth	1	
*Coriandrum sativum* L. **	Apiaceae	Woldimaama	Acidiosis abdominal pain, body swelling	H	L, Fr	F/D	Crushed alone or with or without NaCl, fresh leaves or dry fruits of *R. chalepensis*, fresh rhizomes of *Z. officinale*, and bulbs of *A. sativum* are drunk in a one-to-two-litre concoction through oral administration	14	MH-172
			Blackleg	H	L, Fr	F/D	Crushed with fresh buds of *C. macrostachyus* and F. vulgare, mixed with cold or warm water, drink a one-litre concoction through oral administration	1	
*Crepis rueppellii* Sch. Bip	Asteraceae	Gundi baar adi yoo fiita (fella'i ado)	*Aspiration pneumonia*	H	R	F	Measured with a finger index of the root, chewed and swallowed the juice, spited to the nose	1	MH-277
*Crinum abyssinicum* Hochst ex A. Rich	Amaryllidaceae	Buchchi unkurubba (Goti tuma)	Skin wound	H	R	F	Crushed and pasted on the wound on the dermal	1	MH-274
*Croton macrostachyus* Hochst. ex Delile	Euphorbiaceae	Masana	Actinobacillosis-wooden tongue, FMA	T	Bd	F	Crushed with fresh leaves of *J. procera*, *E. divinorum*, and dry dungs; eat them with warmed *C. macrostachyus* buds with butter; eat their heated or boiled parts, inhaling the mixture smoke; wrap the patient tongue with two to three pieces of dry livestock cattle dung and human hair alive	167	MH-01
			Actinobacillosis-wooden tongue, FMA	T	L		Fresh leaves or buds warmed or heated with leaves of *E. divinorum* and dry dung, then inhaled the smoke (nasal, oral)	1	
			Skin wound	T	La		Fresh buds with buds of *G. auriculiferum*, dry leaf product of *N. tabacum,* and NaCl are pasted on the infected	9	
			Livestock mites, flea and lices (skin ailments /mange mite)	T	L		Crushed with fresh leaves of *C. aurea* and washed the body skin with its concoction	1	
			Anthrax, acidiosis, pasteurellosis	T	Sb, L/Bd		The parts are crushed with fresh root of *S. abyssinica* and dry prepared leaves of *N. tabacum*, mixed with water and butter, and drunk two–three-litre concoct via oral administration	25	
			Acidiosis	T	Sc	D	Powdered with stem charcoal from *E. globulus* and drunk via oral means of administration	1	
			Pasteurellosis, FMD	T	L	F	Fresh leaves crushed, pounded and drunk one litre, and inhale the smoke	2	
			Jaundice	T	L	F	Fresh leaves with fresh leaves of *C. hirsuta* crushed and drunk a one-litre concoction via mouth	3	
			Dingetegna	T	L, Bd	F	Fresh leaves or buds alone or crushed with fresh leaves of *G. amygdalinum* and *E. divinorum* mixed with water and drunk one-two litres; inhale their smoke	7	
			Placenta remain	T	L	F	Fresh leaves with fresh leaves of *G. amygdalinum*, mixed with water, and drunk one-litre concoction	3	
			NCD/A	T	L/Bd	F	Three buds are crushed, pounded with arekie or water, and butter given to the drunk or drunk by syringe	2	
			LSD	T	L, Sb	F	Crushed with dry prepared black *N. tabacum* mixed with NaCl and water drunk one-litre concoction via oral	5	
			Swelling eye, body swelling (Fuxxe'i gana)	T	L, Bd	F	Touch body skin of pained area with warmed boiled/heated fresh leaves/ buds without processed	4	
			Blackleg	T	L, Bd	F	Eat fresh leaves or only three buds with butter; crushed with fresh roots of *M. foetida* and fresh leaves of *H. lusitanica* and *E. divinorum*, drunk one-litre concoct	10	
			Pregnancy (fertility)	T	L	F	Crushed enclosed in fibre; add sex organ for three days	4	
			Abdominal pain	T	Bd	F	Fresh buds crushed and ate with butter		3
			Snake bite (venom injection)	T	L		Crushed, pounded with water and drunk one-litre decoction	2	
			Diarrhoea	T	Sb	F	Crushed fresh leaves of *V. nobilis* and drunk one-litre concoction through oral administration	5	
			Evil eye, evil spirit	T	Rb	F	Crushed, washed the body and drunk one-litre decoction	1	
			Coccidiasis	T	Bd	F	Crushed with fresh two-to-three pods of *C. frutescens*, mixed with butter, and eaten or drunk via oral	3	
			Body swelling	T	Sb	F	Crushed, drunk one-litre decoct; touch the swelled by boiled	2	
*Cucumis ficifolius* A. Rich	Cucurbitaceae	Uulli gereechcho	Rabies, babesiosis, anthrax, blackleg, dinegetegna	Cl	R	F	Crushed fresh roots with or without NaCl or livestock soil salt ('Borra'), mixed in water, and drunk one glass—one litre; also given for dogs and cats, oral and anal	9	MH-14
			Venign external livestock tumour	Cl			Fresh roots are crushed and drunk one-litre decoction via anal or oral administration	2	
*Cyathula uncinulata* (Schrad.) Schinz	Amaranthaceae	Onno’i qaccabba (Gonje'e)	*Aspiration pneumonia*	H	R	F	Crushed, mixed with water, squeezed, and drunk half a litre through the mouth, sniffing or spitting the juice to the nose	9	MH-199
			Blackleg	H	R	F	Crushed, mixed with water and drunk one-litre decoction for every morning for four days	3	
*Cymbopogon citratus* (DC.) Stapf. **	Poaceae	Hixaana	Conjunctivitis/eye pain	H	R, L	F	Chewed and spited; crushed and squeezed; add two up to three drops by syringe in the morning and night	39	MH-86
			*Aspiration pneumonia*	H	L	F	Crushed, mixed with water, drunk one litre, inhale, chewed, and swallowed the juices by oral means	2	
			Bloat	H	L	F	Crushed and drunk one-litre decoction through mouth	1	
*Cyperus rotundus* L	Cyperaceae	Naaqa	Acidiosis	G	R	F	Crushed, mixed with water and drunk one coffee cup or one glass-one litre of decoction via mouth	2	MH-168
*Cyphostemma adenocaule* (Steud. ex A. Rich.) Desc. ex Wild & R.B. Drumm	Vitaceae	Dodoobba (Jaanjeechcho)	Acidiosis body swelling, snake bite	Cl	R	F	Crushed and drunk half a coffee cup—one litre of the decoct	1	MH-339
*Cyphostemma pannosum* Vollesen	Vitaceae	Gidiidoola	Skin wound	H	R	F	Roots are crushed, mixed with water, and pasted on the wound	9	MH-330
			Acidiosis blackleg, telleriosis (anaplasmiosis), spleen enlargement-tick-borne ailment, retained placenta, anthrax	H	R	F	Crushed and drunk one glass to two litres of decoction through oral and anal administration	4	
*Datura stramonium* L.**	Solanaceae	Machaa’l haqqa	Rabies	H	R	F	Crushed with *S. abyssinica* roots or only mixed with water and drunk one glass or mug—two litres—one ‘Bulle’e’/equal to one Pepsi bottle’(Had.) immediately via mouth or anus	10	MH-69
*Dicliptera foetida* (Forssk.) Blatt	Acanthaceae	Omoro'o laba	Bat urine ailment	H	L	F	Crushed, mixed with water, and drunk one litre, washing the body with the decoction	1	MH-136
*Dicliptera magaliesbergensis* K. Balkwill	Acanthaceae	Baxaaxursa/Omoro labi jule'i/mani illi qaraare)	Pasteurellosis, evil eye (evil spirit)	H	R, L	F	Crushed, mixed with water, and drunk one litre of decoction via mouth while washing the body skin	1	MH-158
*Dodonaea viscosa* subsp. *angustifolia* (L.f.) J.G. West	Sapindaceae	Kitkiita	PPR (Peste des petits ruminants), diarrhoea	S	Bd	F	Fresh leaves with buds of *G. amygdalinum*, buds of *C. macrostachyus*, *E. depauperata*, *B. antidysenterica*, *R. cordifolia,* *J. chimperiana, S. elliptica*, and *P. dodecandr*a, mixed with water, and drunk half a glass for young calf and two glasses for adults	17	MH-19
			Bat urine, back sore	S	L	F	Crushed, pasted on the painful area or wound, and tied	2	
			Bloat	S	L	F	Crushed and drunk one-litre decoction	1	
*Echinops kebericho* Mesfin. *	Asteraceae	Toosa	Abdominal pain Acidiosis	H	R	F	Crushed, mixed with water, and drunk one glass up to one litre of oral and anal administration	2	MH-195
*Ekebergia capensis* Sparrm	Meliaceae	Oloola	Rabies	H	Sb	F	Crushed the fresh leaves of *Z. asiaticum*, the fresh stem bark of *S. oxyacanthum*, and *A. falcatus*, mixed them with water, and drunk three coffee cups of the concoction	4	MH-248
*Ensete ventricosum* (Welw.) Cheesman. *	Musaceae	Weesa	Placental remain, trauma (broken bones)	H	L, Ps, Cm	F	Fresh red leaf/pseudo stem/corm ('Hamicho'-Had.) roasted and eaten until healed or removed from the remained placenta orally	21	MH-20
			Acidiosis, diarrhoea	H	R	F	Crushed or ‘Bu’o-Had’, ‘Hamicho-Had.’, mixed with water, and drunk one litre of corm ('Hamicho'-Hadiyissa) eaten livestock, eaten roasted/cooked corm	9	
			Toxicity	H	Ps	F	Freshly crush, squeeze water, and drunk two litres of oral	1	
*Eragrostis tef* (Zucc.) Trotter. *	Poaceae	Xaafe’e	Sore	H	Se	D	Unprocessed seeds are mixed with donkey faeces and pasted on the sore wound	1	MH-223
*Erica arborea* L	Ericaceae	Saate'e	Constipation	S	Bd	F	Fresh buds or leaves are crushed; drunk one litre orally	1	MH-233
*Erythrina abyssinica* Lam	Fabaceae	Qaala’i wora’a	Acidiosis	T	Sb	F	Crushed, mixed with water, and drunk one litre of the decoction and given oral administration	2	MH-332
*Erythrina brucei* Schweinf. *	Fabaceae	Wora’a	*Aspiration pneumonia,* acidiosis, constipation, abdominal pain	T	Sb	F	Chewed with fresh stem bark of *D. schimperiana* and/or with dry leaves of *N. tabacum* spitted to the nose, drunk one litre of concoction by oral and nasal route	15	MH-55
*Eucalyptus globulus* Labill. **	Myrtaceae	Qadaalli baarzaafa	Bloat	T	Sc	D	Drunk one litre of charcoal from *C. macrostachyus* and *H. lusitanica* mixed with water, and drunk one litre of concoct	11	MH-54
			Dingetegna	T	Bd	F	Boiled, mixed with butter, and eaten; inhaled the smoke	2	
			Parasitic leech	T	L	F	Crushed with fresh *C. esculenta* rhizome and drunk	2	
			Coughing, infection of grass hopper entrance, listeriosis	T	L	F	Crushed with dry prepared leaves of *N. tabacum*, mixed with water, and drunk one litre in three alternative days until healed	4	
*Euclea divinorum* Hiern	Ebenaceae	Meegaara	Actinobacillosis, blackleg, dingetegna	T	Bd	F	Crushed with fresh buds of *C. macrostachyus*, boiled and eaten; burned with dry livestock dungs inhaled the smoke through the nose, oral, and nasal	16	MH-15
			Bloat	T	L	F	Fresh buds are crushed with fresh buds of *R. neglecta* and *J. procera*, mixed with water, and drunk one litre	1	
			Diarrhoea, acidiosis	T	L	F	Crushed alone or with fresh root of *B. suaveolens,* mixed with water, and drunk one litre	2	
			Conjunctivitis/eye ailment	T	Bd	F	Chewed and spitted to the eye	2	
*Euphorbia abyssinica* J.F. Gmel	Euphorbiaceae	Adaamma	Asthma (stenosis, respiratory organ)	T	St-Br	D	Dry stem branch burning and inhaling the smoking gas, nasal and oral administration	15	MH-200
			Body swelling, wound, livestock tumour	T	La	F	Milky latex juice extract and NaCl are added, then smeared on the cutted skin wound and swollen part; it burst the swell and released out as pus	6	
*Euphorbia depauperat*a Hochst. ex A. Rich	*Euphorbiaceae*	Gendeella	PPR (Peste des petits ruminants)	H	Bd/L	F	Crushed, mixed with water, and drunk one glass of young calf, two glasses of adults, orally	1	MH-47
			LSD	H	Bd/L		Crushed, mixed with water, and drunk one litre of decoction oral administration	4	
*Foeniculum vulgare* Mill	Apiaceae	Wollanga (Ashbe’e)	*Aspiration pneumonia*	H	L, Fr	F	Fresh leaves and fruits are chewed and spitted into the nose	4	MH-66
			Blackleg, pasteurellosis	H	L, Fr	F	Crushed, powdered, mixed with water, and drunk one litre of decoction, oral	1	
*Grevillea robusta* A. Cunn. ex R.Br. **	Proteaceae	Giraar shuwwishuwwa	Constipation, diarrhoea, LSD, Acidiosis	T	L	F	Crushed alone or with fresh leaves of *S. hypselodendron*, mixed with water, drunk one litre	5	MH-106
*Gymnanthemum amygdalinum* (Delile) Sch.Bip	Asteraceae	Heebbaa	Actinobacillosis, trauma/blood accumulation, PPR (Peste des petits ruminants), diarrhoea, babesiosis (liver ailment), dinegetegna, acidiosis, placenta remain	T	L, Bd	F	Crushed and mixed with water, and drunk one litre of decoction for adults and half a litre for young people via oral administration	19	MH-07
			Conjunctivitis/eye pain	T	L	F	Mixed with water, squeezed, filtered, and added three drops to the eyes	3	
*Gymnanthemum auriculiferum* (Hiern) Isawumi	Asteraceae	Baarawwa	Wound	S	L, Bd	F	Crushed with fresh/dry leaves of *N. tabacum*, pasted and tied; added the juice until cure	11	MH-03
			*Fowl typhoid*	S	L, Bd	F	Crushed the fresh or dry leaves of *N. tabacum* and drunk the juice through the beak via the oral route	5	
*Gymnanthemum* sp.	Asteraceae	Aggagga	Dingetegna, diarrhoea, trypanomiasis	S	R	F	Crushed, mixed with water, and drunk two-litre infusion of decoctions by oral administration	3	MH-309
*Gymnosporia arbutifolia* (Hochst.ex. A. Rich.) Loes	Celastraceae	Jonge’e	Conjunctivitis (eye pain)	T	R	F	Crushed, mixed with water, filtered by a neat white cloth, and added two to three drops to the eyes	1	MH-126
			*Aspiration pneumonia*	T	R	F	Chewed fresh roots spitted to the nose, nasal route	1	
*Hagenia abyssinica* (Bruce) J. F. Gmel	Rosaceae	Suuxo	Bat urine ailment	T	Fl, Se	D	Crushed and mixed with water and drunk 1 coffee cup 1st by nose and ear; 2nd 1itter by anus at morning	1	MH-173
*Helianthus annuus* L.**	Asteraceae	Faaranj nuuga	Body swelling	H	Se	D	Powdered and mixed in water, and drunk one litre	1	MH-183
*Hesperocyparis lusitanica* (Mill.) Bartel. **	Cupressaceae	Faaranj hooma	Livestock trypanosomiasis, blackleg, dingetegna	T	L	F	Crushed fresh leaves NaCl, mixed with water and drunk one litre via mouth or anus	6	MH-61
*Hordeum vulgare* L.**	Poaceae	So'o	Acidiosis	H	Se	D	Powdered burned seeds, mixed them with water, and drunk one-litre solution of decoction orally	1	MH-222
*Hymenodictyon floribundum* (Hochst. & Steud.) B.L. Rob	Rubiaceae	Odeera (Wo'l qobbo'o)	Cysticercus: bovis, ovis (livestock ascariasis)	S	L	F	Leaves are crushed, mixed in water, and drunk. half-litre decoction given orally	1	MH-334
*Ilex mitis* (L.) Radlk	Aquifoliaceae	Ashmiinqa	Acidiosis	T	L	F	Crushed with fresh leaves or fruits of *C. sativum*, bulbs of *A. sativum,* and NaCl, drunk one litre and drunk one litre	2	MH-241
*Juniperus procera* L	Cupressaceae	Abash hooma	Actinobacillosis (wooden tongue)	T	L	F	Fresh leaves boiled or burned in the fire with *C. macrostachyus* and smoked with the steam or gas	1	MH-60
*Justicia schimperiana* (Hochst.ex. Nees) T. Anderson	Acanthaceae	Xummunga	FMD, diarrhoea	S	Bd		Fresh buds were boiled with fresh buds of *C. macrostachyus*, breathing or inhaling the smoke through the nose or mouth, crushed, and drunk one mug for calf and two mugs for adults with water	2	MH-31
*Kalanchoe hypseloleuce* Friis & M.G. Gilbert. *	Crassulaceae	Hancuura	Body swelling	H	L	F	Boiled fresh leaves or stems in fire, crushed, and inserted in the painful area by cutting the painful place	1	MH-127
*Lasiosiphon glaucus* Fresen	Thymelaeaceae	Ollawwa	FMD	S	Sb	F	Stem bark is pressed, tied to the head and legs, and crushed water infusions of decoctions via oral route	46	MH-37
			Rabies	S	Sb	F	Crushed stems from sunrise and drunk one litre of decoction	5	
*Lathyrus oleraceus* Lam. **	Fabaceae	Gite'e	Nasal bote	H	Wh	D	Crushed dry straw with dry *N. tabacum* leaves, mixed in their drinking water, and killed them in the water	1	MH-227
*Lavandula dentata* L	Lamiaceae	Qadaalli wereeggi fiita (Naatira laba)	Abdominal pain/ache	H	L	F	Crushed with NaCl, mixed with water, and drunk one litre	1	MH-167
*Lysimachia ruhmeriana* Vatke	Primulaceae	Uulli saratichcho (Guffi saratichcho)	*Aspiration pneumonia*	H	R	F	Roots chewed with NaCl and spitted to the nose	1	MH-139
*Maesa lanceolata* Forssk	Primulaceae	Kowwaada	Babesiosis, diarrhoea, aspiration pneumonia	T	Sb	F	Crushed from sunrise, mixed in cold or warm water, juice add to nose; drunk one mug-one litre; spitted to the nose; oral and nasal administration	5	MH-02
*Melia azedarach* L. **	Meliaceae	Niima laba	Dingetegna	T	L	F	Crushed, mixed with water, and drunk in one litre of decoction; orally	4	MH-209
*Millettia ferruginea* (Hochst.) Hochst. ex Baker. *	Fabaceae	Billawwaqqa	Coughing, *aspiration pneumonia*	T	Sb, L	F	Crushed the fresh leaves of *D. viscosa* subsp*. angustifolia*, mixed them with water, and drunk one-litre of the concoct; orally	3	MH-97
							Chewed and spitted to the nose or mouth	2	
*Momordica foetida* Schumach	Cucurbitaceae	Hamash waasa	Diarrhoea	Cl	R	F	Crushed, mixed with charcoal powdered with *C. molle* in cold or warmed water; drunk one to two litres, young to adults	48	MH-06
			Abdominal pain, abdominal ache	Cl	R	F	Crushed fresh roots and leaves of *S. hypselodendron*, mixed in cold or warmed water, drunk one litre or powdered mixed in water and drunk one litre separately	3	
			Bat urine ailment, *aspiration pneumonia*, acidiosis	Cl	L, R	F	Crushed, mixed cold or heated water, added the juice to the urine-injured area, and drunk one litre with fruits or leaves of *C. sativum* for bloating via anal administration	13	
			Blackleg, dingetegna, anthrax	Cl	R	F	Crushed roots alone or with leaves of *C. macrostachyus* mixed with cold or warmed water, and drunk one-two litres of oral	16	
			Telleriosis/anaplasmiosis (spleen enlargement-tick-borne ailment)	Cl	R	F	Crushed with dry prepared leaves of *M. foetida*, mixed with two cups local arekie, and drunk one litre of concoction	3	
			LSD	Cl	L	F	Crushed with dry leaves of *N. tabacum* and bulbs of *A. sativum*, drunk one litre until healed via mouth	3	
			Swelling oxen head area due to fraction of yoke	Cl	L	F	Fresh leaves crushing and rubbed on the pained area	1	
			*Epizootic lymphangitis*	Cl	L	F	Fresh leaves are crushed and drunk in one litre via anal administration; orally	1	
			Actino-bacillosis (cause wooden tongue)	Cl	L	F	Crushed with fresh leaves or buds of *C. macrostachyus*, drunk one litre; eat boiled buds of it; and inhale the smoke with dry dung	2	
			Evil eye (evil spirit)	Cl	R, L	F	Crushed freshly and drunk one litre immediately, oral	1	
*Myrtus communis* L	Myrtaceae	Goonchi qasha'a	Diarrhoea	S	Sb	F	Crushed from sunrise; drunk one litre until cured, oral	1	MH-169
*Nicotiana tabacum* L.**	*Solanaceae*	Tambaa'i koshsho'o	LSD, Acidiosis, Coughing, livestock TB, body swelling	S	L	FD	Crushed, fresh, or dry black product by *E. depauperata* fresh leaves alone or with a bulb of *A. sativum*, rhizome of *Z. officinale* fresh leaves of *C. macrostachyus*, mixing with water, and drinking one to two litres for two days via mouth and anus	162	
			*Aspiration pneumonia*	S	L	D	Dry prepared leaves with sunrise stem bark of *E. brucei* and *A. schimperiana* chewed and spitted the fluid to the nose; closed with fibre chewing and spitting juice	17	
			Skin wound	S	L	D	Dry leaves crushed with NaCl, powdered with fresh buds with latex of *C. macrostachyus* and *G. auriculiferum*, mixed with water, paste; tied	22	
			Back sore	S	L	D	Crushed, powdered and sprinkled on the painful area	12	
			Nasal bote, snake bite, insects’ infection	S	L	D	Crushed with a bulb of *A. sativum* and a rhizome of *Z. officinale*, covered with ensete fibre, held in the mouth and spitted to the nose or mouth, crushed, and sprayed to protect the snake from a bite on the physical environment	28	
			Body swelling, telleriosis	S	L	D	Crushed with rhizome of *Z. officinale*, mixed with local arekie, and drunk one litre, rubbed swollen area	11	
			Conjunctivitis (eye pain)	S	L	D	Crushed, squeezed, and added three drops of the filtrate, then added two–three drops by syringe	6	
			Livestock tumour	S	L	D	Crushed dry prepared leaves, mixed with water paste and tied on wounded area; drunk one-litre charcoal	2	
			PPR (Peste des petits ruminants)	S	L	D	Crushed and pasted the decoction on the sore mouth	3	
			NCD	S	L	D	Crushed and mixed with arekie and drunk by using a syringe through the beak, oral means	4	
*Nigella sativa* L.**	Ranunculaceae	Heemachchi enja	Acidiosis	H	Se	D	Crushed with dry or fresh leaves and dry of *C. sativum*, *A. corrorima*, and *R. chalepensis*, rhizome of *Z. officinale*, and A. sativum, powdered, mixed with water, and drunk one litre through the mouth and anus	5	MH-03
*Ocimum basilicum* L.var. *cinnamon* Basil sweet. **	Lamiaceae	Gimmenja (Basso'i bila laba)	Constipation	H	L	F	Crushed and drunk one litre of decoction via oral administration	1	MH-148
*Ocimum lamiifolium* Hochst.ex Benth	Lamiaceae	Minaantoofa	Acidiosis (bloat)	H	L, Ag	F	Crushed with livestock salt ('Borra') and fresh or dry bulbs of *A. sativum*, *Z. officinale*, and *R. chalepensis*, drunk one litre of concoction via mouth	4	MH-23
*Ocimum spicatum* Deflers	Lamiaceae	Buubayye (Angaambiisha)	Conjunctivitis (eye pain)	S	L	F	Crushed and added two–three drops by syringe	5	MH-67
			LSD, michi, African horse sickness/AHS	S	L	F	Crushed alone or with fresh leaves of *N. tabacum*, mix and drunk one glass of concoction by oral route	1	
*Oldeania alpina* (K. Schum.) Stapleton	Poaceae	Leema	Trauma (broken bone)	H	St	D	Dry stems are chopped or pressed and tied to the broken bone for proper attachment	1	MH-174
*Olea europaea* L. subsp. *cuspidata* Wall. ex G. Don	Oleaceae	Weera	Livestock tumour	T	Bd	F	Fresh buds are crushed and drunk one-litre concoction through oral administration	2	MH-111
*Olea welwitschii* (Knobl.) Gilg & G. Schellenb	Oleaceae	Siigeeda	*Aspiration pneumonia*	T	Sb, L	F	Fresh stem bark from sunrise, fresh leaves crushed and spitted to the nose	2	MH-261
*Olinia rochetiana* A. Juss	Penaeaceae	Guna	*Aspiration pneumonia*	T	Bd	F	Fresh buds are crushed and spitted into the nose	2	MH-39
			Pregnancy (fertility)	T	Bd	F	Crushed with fresh buds of *C. macrostachyus* and *C. longicauda*-enclosed fibre and inserted in the female sex organ for 30 min	1	
*Oncoba spinosa* Forssk	Salicaceae	Itakkam kuukka	Abdominal pain	T	L, Fr	F	Fresh leaves or ripe raw fruits crushed and drunk one litre	1	MH-351
*Oxalis corniculata* L.**	Oxalidaceae	Goro’ama (cii'i mixmimixo'o)	Snake bite (venom injection)	H	L	F	Crushed, and drink one mug, jug, or litre of the solution decoction through the mouth	1	MH-350
*Pavetta oliveriana* Hiern	Rubiaceae	Gaarawwa laba (Meentichchi gaarawwa)	Bat urine ailment	S	L	F	Crushed and drunk one litre; dry faeces of eagle powdered and drink one-litre decoction	1	MH-228
*Pentanema confertiflorum* (A. Rich.) D.Gut.Larr., Santos-Vicente, Anderb., E. Rico & M.M. Mart.Ort. *									
	Asteraceae	Anca (qadaalli haagallo'o/Bulshaana laba)	Coughing Pasteurellosis	S	L	F	Fresh leaves are crushed, pounded in water, and drunk one litre; NaCl and livestock soil salt (‘Borra’) are drinking one litre orally	2	MH-300
*Peponium vogelii* (Hook.f.) Engl	Cucurbitaceae	Humbusha (Dunguruulla)	Anaplasmiosis (spleen enlargement)	H	L	F	Crushed, fresh leaves and drunk half litre of water mixed decoction via mouth	1	MH-242
*Phaseolus lunatus* L	Fabaceae	Boloqe’e (Lob otongora)	Dingetegna Diarrhoea	Cl	R	F	Crushed, mixed in cold or warm water, and drunk one-two litre solution for equines (mule, donkey, and horse)	2	MH-216
*Phoenix reclinata* Jacq	Arecaceae	Sale’e (Dimbaaba)	Conjunctivitis (eye pain)	T	Se, Bd, Tw		Chewed either the parts and spitted a drop; add two- three drops into the painful eye	4	MH-110
*Phyllopentas schimperi* (Hochst.) Y.D. Zhou & Q.F. Wang	Rubiaceae	Wo’l oda'a	Cysticercus, bovis, ovis/ascariasis	H	L	F	Crushed, mixed with milk, and drunk one litre of decoction through oral administration	1	MH-232
*Physalis peruviana* L	Solanaceae	Onjooro’o	Dingetegna	H	R	F	Crushed, mixed with water, and drunk one litre via mouth	2	MH-114
*Phytolacca dodecandra* L’Her	Phytolaccaceae	Haanja	*Aspiration pneumonia*	S	L, R	F	Chewed three leaves, buds, and roots, squeezed, and spitted the filtered water to the nose	10	MH-161
			Diarrhoea	S	Bd	F	Crushed and drunk two mugs for adults, half for calves	6	
*Piliostigma thonningii* (Schumach.) Milne-Redh	Fabaceae	Maccoqaara (Qaala'i weddeeshsha)	Bloat	T	L	F	Fresh leaves are crushed and drunk in one-litre decoction by oral administration	1	MH-323
*Platostoma africanum* P. Beauv	Lamiaceae	Heedoo'l maaxa	Diarrhoea	H	L	F	Crushed and mixed it with water and drunk one-litre decoction through oral means	2	MH-25
*Prunus africana* (Hook.f. Kalkm.)	Rosaceae	Araara	Body swelling, bloat	T	Sb	F	Fresh stem bark from sunrise was crushed, mixed with cold water, and drunk one-litre solution	3	MH-57
			Back sore	T	Sb	F	Crushed from sunrise, paste on the wound	1	
*Ricinus communis* L	Euphorbiaceae	Qobbo’o	Swelling eye, body swelling	S	L	F	Boiled or warm and touch the painful body skin	1	MH-125
*Rotheca myricoides* (Hochst.) Steane & Mabb	Lamiaceae	Haniga	*Aspiration pneumonia,* evil sprit	S	Sb, L. Bd	F	Chewed sunrise-stem bark or buds or leaves and spitted the juice to the nose.; stem bark or either part is amulated. on the head or on the hind leg of cattle to against evil eye/sprit	4	MH-186
*Rubia cordifolia* L	Rubiaceae	Haaro’o (Baarxusha)	Michi diarrhoea, *aspiration pneumonia,* bat urine ailment	Cl	L, R	F	Crushed roots alone or with buds of fresh *D. viscosa* subsp. *angustifolia* leaves, mixed with water, and drunk one litre; spitted to nose the decoction via mouth	8	MH-68
*Rumex abyssinicus* Jacq	Polygonaceae	Shiisho’o	Babesiosis, dinegetegna, abdominal pain	H	R	F	Crushed, pounded in water, homogenized in cold water, and drunk a litre of decoction via oral route	19	MH-22
			Pregnancy (fertility)	H	Bd	F	Crushed with three fresh buds of *C. macrostachyus*, fresh *C. schimperi* roots, enclosed fibre and insert in female sex organ, and tied lower tip at the tail	3	
			Livestock tumour (skin wart/venign-external tumour)	H	R	F	Crushed, pasted, and then tied to the painful area	5	
*Rumex nepalensis* Spreng	Polygonaceae	Go’ichcho	Rabies	H	R, L	F	Crushed it, mixed it with water, and drunk one glass or mug decoction via mouth	3	MH-21
*Ruta chalepensis* L. **	Rutaceae	Qantalaama	Abdominal pain, acidiosis (bloat)	H	L, Fr	F/D	Crushed with fresh or dry leaves or fruits of *C. sativum*, seeds of *A. corrorima*, rhizome of *Z. officinale*, and bulbs of *A. sativum* mixed with NaCl or salt soil of livestock (‘Borra’), soil in cold water, and drunk one-two litres	99	MH-83
			Blackleg	H	L, Fr	F/D	Fresh leaves and fresh or dry fruits are crushed and drunk one-litre decoction by oral or anal administration	5	
*Rytigynia neglecta* (Hiern.) Robyns	Rubiaceae	Garawwa	Acidiosis	S	Bd	F	Crushed together with the fresh buds of *E. divinorum* and fresh buds of *J. procera*, mixed and drunk	1	MH-48
*Scadoxus multiflorus* (Martyn) Raf	Amaryllidaceae	Got tuma (Hamashshi weesa)	Acidiosis	H	R, L	F	Crushed the dry-prepared *N. tabacum* leaf and drunk a litre of concocted solution	3	MH-238
			Shoat pox	H	R	F	Crushed, mixed with water, and drunk one coffee cup decoction via mouth	2	
*Schrebera alata* (Hochst.) Welw	Oleaceae	Lob haqqa	Conjunctivitis (eye pain)	T	L	F	Squeezed the crushed, mixed with water, filtered, and added three drops to the eyes or spitted to the nose	1	MH-284
*Securidaca longepedunculata* Fresen	Polygalaceae	Mukke'e	Dinegetegn acidiosis, babesiosis. anthrax, body swelling abdominal pain, actinomycosis	T	Rb	F, D	Crushed its part from sunrise, mixed with water, and drunk one litre of decoction oral and anal administration	25	MH-206
			Diarrhoea	T	Rb	F, D	Crushed, with root bark of *X. americana* and stem bark of *C. molle*, mixed water, and drunk one litre of concoct	7	
*Shirakiopsis elliptica* (Hochst.) Esser	Euphorbiaceae	Shaqama	Bat urine ailment, diarrhoea	T	L	F	Crush fresh leaves and apply cream or ointment to the wound	1	MH-33
*Sida rhombifolia* L	Malvaceae	Qarxaffa	Snake bite (venom injection), acidiosis, constipation abdominal pain	S	L	F	Crushed and mixed it with water and drunk a mug to one-litre decoct via mouth	5	MH-134
*Spiniluma oxyacantha* (Baill.) Aubrév	Sapotaceae	Faraxxi qasa	Rabies	T	Sb	F	Crushed and drunk three coffee cups of decoction for one day via mouth or anus	4	M-28
*Solanum incanum* L	Solanaceae	Heemachchi looraawwa	Actinomycosis	S	Fr	F	Ripe fresh fruits, crush, decoct, and drunk. half litre	3	MH-38
			Conjunctivitis (eye pain)	S	Fr	F	Ripe fruit was crushed, squeezed, mixed with water, and added to the eyes with two–three drops	1	
			Bloat	S	R	F	Crushing fresh roots and drunk one mug / a glass orally	2	
			Actinobacillosis (wooden tongue)	S	Fr	F	Fresh, ripe fruits are crushed and given with butter	2	
*Stephania abyssinica* (Quart. -Dill. & A. Rich.) Walp	Menispermaceae	Huma	Diarrhoea, acidiosis, anthrax, pasteurellosis, dingetegna	Cl	R	F	Crushed with fresh stem bark from the sunrise of *C. macrostachyus* and drunk 1 mug of concoct via mouth	30	MH-49
			Rabies	Cl	R	F	Crushed fresh leaves of *D. stramonium* and drunk one litre of concoction through the mouth and anus	7	
*Tapinanthus* sp.	Loranthaceae	Buni xanqo	LSD	Hemp	L	F	Crushed fresh whole parts and drunk 1 L of decoction by means of oral route	1	MH-202
*Terminalia brownii* Fresen	Combretaceae	Dibi'n haqqa	Constipation	T	Sb	F	Fresh stems are crushed and drunk in one litre of decoction by mouth	1	MH-327
*Thymus schimperi* Ronniger. *	Lamiaceae	Ishina	*Aspiration pneumonia*	H	L	F/D	Chewed and spitted at the nose or mouth of sick livestock through the oral or nasal route	1	MH-85
*Toddalia asiatica* (L.) Lam.(Synonym of: *Zanthoxylum asiaticum* (L.) Appelhans, Groppo & J.Wen.	Rutaceae	Seego'o	Rabies	Cl	L	F	Crushed fresh leaves, mixed with water, and drunk one mug until cure	2	MH-09
			Dingetegna	Cl	Fr	F	Crushed with fresh leaves of *C. macrostachyus* and *E. divinorum*, drunk one litre of the infusion of concoct	2	
*Trigonella foenum- graecum* L. **	Fabaceae	Shuqoota	Faciolosis	Cl	Se	D	Mix poweder seeds with water and drunk one litre for bovines, sheep, and goats via mouth	1	MH-94
*Urera hypselodendron* (Hochst. ex A. Rich.) Wedd. (Synonym of: *Scepocarpus hypselodendron *(Hochst. ex A.Rich.) T.Wells & A.K.Monro.	Urticaceae	Hariira (Dooqa)	Cysticercus, bovis, ovis (ascariasis), acidiosis, constipation,	Cl	L	F	Crushed alone or with fresh leaves of *G. robusta*, mixed with water, and drunk one litre of water solution, oral	13	MH-130
*Urtica simensis* Hochst. ex A. Rich*.* *	Urticaceae	Amaa'l doobba (Cimcima)	Evil eye (evil spirit)	H	L, Bd	F	Crushed with fresh roots of *A. africanus*, mixed with water, and drunk one litre, concoction, oral mean	3	MH-194
			Bat urine ailment	H	L	F	Crushed, mixed with *A. dimidiata*, and drunk three mug infusion concoction	3	
*Vepris nobilis* (Delile) Mziray	Rutaceae	Xaa'a	Acidiosis	T	L	F	Crushed, mixed with water, and drunk one litre of decoction via mouth	2	MH-73
			Diarrhoea, constipation	T	Sb	F	Crushed from sunrise both with *C. macrostachyus* and drunk one litre of concoct via mouth	2	
*Verbascum sinaiticum* Benth	Scrophulariaceae	Got buyya	*Aspiration pneumonia*	H	R	F	Chewing and spitting at the nose decoction	4	MH-316
			Constipation	H	L	F	Crushed, mixed with water, drunk one -two litre decoct	5	
*Verbena officinalis* L	Verbenaceae	Qisqisa (Modollo'o)	Abdominal pain	H	R, L	F	Roots or leaves are rushed, and a litre of decoction is drunk via mouth	5	MH-166
*Withania somnifera* (L.) Dunal	Solanaceae	Ajaar buyya	Arthritis (gouge or mondy-morning ailment), acidiosis	S	L, R	F	crushed fresh leaves or roots, mixed them with water, and drunk a one-litre decoction for bovines and equines via oral and anal administration	4	MH-378
*Ximenia americana* L	Olacaceae	Qaala’i kooshshaama	LSD, diarrhoea, *aspiration pneumonia*	S	L, Sb	F	Crushed, mixed with water, and drunk one litre until healed; spitted the decoction to the nose via oral and nasal means	4	MH-273
*Zea mays* L.**	Poaceae	Boqqolla	Acidiosis	H	Se	F	Powdered burned seed, mixed with water, and drunk one litre	225	MH-225
*Zingiber officinale* Roscoe. **	Zingiberaceae	Jaanjibeela	Actinomycosis, acidiosis, abdominal pain, diarrhoea	H	Rh	F	Crushed the bulbs of *A. sativum,* dry or fresh leaves or fruits of *C. sativum*, *R. chalepensis,* and dry seeds of *A. corrorima* and drunk the concoction	64	MH-64

Growth form: Habit: Hàb = habit; H = herb, S = Shrub, T = Tree, Cl = Climber, and Hemp = Hemi-parasite; Part used = PU (Whole part = Wh; above ground = Ag or if below ground = Bg); Leaf = L; Root = R; Stem = St; Flower = Fl; Fruit = Fr; Pod = Pd; Seed = Se; Rhizome = Rh; Bulb = Bu; Bark = Ba, Stem bark = Sb, Stem charcoal = Sc, Root bark = Rb; Latex = La, Buds = Bd, Bula = Bl, Pseudo stem = Ps, Corm-Cm); Conditions of the Preparation = CP (Fresh-F; Dry-D; Fresh or Dried- F/D); Preparation Forms (PF), MT, and Ra are used symbols or as it = PF (Burning = Bn, Chewing = Ch, Concoction = Co, Decoction = De, pounded = Pu, powdered = Po, or Grinding = Gr, Crushing = C, Warming/Boiling/Heating = Wa/Bo/He, Roasted/Cooked = Ro/Ck, Infusion = Inf, Squeezing = Sq, Cutting = Cu, Chopping = Cp); Means of Treatment = MT(Drinking = Dk, Smoking = Smo, Eating = Et, Fumigating = Fum, Ho = Holding on pained tooth, Touch the pained area = Tu, Inhaling/sniffing = In/Sn, Inserting = Ins, Pasting = Pa, Painting/Creaming/Smearing = Pt/Cr/Sm, Dermal/External = Dr/Ex, Spitting to the mouth or eye = Sp, Sprinkled = Spr, Rubbing = Ru, Dropping = Dp, Eye = Ey, Swallowing the chewed juice = Sw, Amulet = Am a plant part which is tied at legs or on the horn/head of cattle and legs, Spraying, = Spy, Tie around the pained area = Td,Wrapped = Wr, Without process = Wp; Smelling through nose/mouth = Sme, Washing = W); Physical = Ph; and Route of Administration = RA (Oral = O; Nasal = N; Eye (Optical) = Ey/Op; Dental = Dt; Dermal = Dr, Ear (Auricular) = (E/Au), Anal = An, and, Endemic to Ethiopia (*), Introduced into Ethiopia (**), Native not used asterisks, Total number of informants who cited the medicinal plants for treating the major aliments = UR ( use report); VN: = Vernacular name, and MH = Mulatu Hankiso

*Croton macrostachyus* was used to treat 13 ailments (3.99%): FMD, blackleg, bloat, pregnancy and actinobacillosis of bovines; abdominal pain and abdominal ache of bovines and sheep; livestock tumour of bovines and equines; used to treat all livestock diarrhoea, livestock hepatitis/jaundice, dingetegna, snake bite and skin wound; both *Allium sativum* and *Gymnanthemum amygdalinum* plant species (0.75% each) used to treat nine ailments (2.76% each): the former was used to treat NCD of poultry; bovines LSD, blackleg and wooden tongue; anaplasmiosis of bovines and sheep; constipation bovines, sheep and goat; dingetegna, bloat, parasitic leech and coughing of all livestock; whereas the latter also was used to treat PPR (peste des petits ruminants) of sheep and goats; diarrhoea of bovines, sheep and goat; placenta remain of bovines, sheep, goats, horses and donkeys; babesiosis (liver ailment), eye pain, dingetegna, bloat and trauma (blood accumulation in the body) of all livestock; and actino-bacillosis/wooden tongue of bovines.

*Securidaca longepedunculata* was used to treat eight ailments (2.45%), anthrax of bovines, equines, sheep; actinomycosis of bovines; diarrhoea of bovines, sheep and goat; dingetegna, abdominal pain/ache, bloat, swelling, and babesiosis of all livestock were treated. Both *Cucumis ficifolius* and *Zingiber officinale* plant species (both treat 1.51%) were used to treat seven ailments (2.15% each); among these, *C. ficifolius* was used to treat mainly blackleg of bovines; anthrax of bovines, sheep and equines; external tumour of bovines and equines; and all livestock of babesiosis, rabies, abdominal pain/ache, and dinegetegna were also treated; whereas *Z. officinale* was used to treat livestock ailments of swelling, actinomycosis, and LSD of bovines; diarrhoea of bovines, sheep, and goat; coccidasis of poultry ailment; bloat and abdominal pain/ache of all livestock were treated in the study area.

The other three plant species (2.27%) that were used to treat six (1.84% each) individual ailments: *Cyphostemma pannosum* was used to treat skin wound and bloat of all livestock; placenta remains in bovines, sheep and goats; blackleg in bovines; telleriosis (spleen enlargement) in bovines and sheep; anthrax in bovines, sheep and equines. *Eucalyptus globulus* was used to treat listeriosis, dingetegna, bloat, nasal bote and coughing in all livestock; it was also used to treat insect infections in bovines, sheep and goats. *Euclea divinorum* was used to treat actinobacillosis of bovines, conjunctivitis, bloat, dingetegna and diarrhoea of all livestock, and blackleg of bovines.

Four plant species (3.03%) were used to treat five ailments (1.53% each), such as *Capsicum frutescens* was used to treat poultry coccidiasis and NCD in chickens; spleen enlargement in bovines and sheep; LSD and livestock TB in bovines; *Ensete ventricosum* was used to treat placental remain of bovines, sheep and goats; diarrhoea, bloat and trauma (broken bones) of all livestock; and toxicity in poisoned livestock of bovines, sheep and goats*; Solanum incanum* was used to treat actinobacillosis and actinomycosis of bovines; bloat, swelling and conjunctivitis of all livestock; *Stephania abyssinica* was used to treat bloat, diarrhoea and rabies in all livestock; anthrax in bovines, sheep and equines; and pasteurellosis in bovines.

Among the lower number of ailment-treating plant species, nine plant species (6.82%) were used to treat four ailments (1.23% each): for example, *Calpurnia aurea* was used to livestock mites, fleas and lice in bovines*;* skin ailment in all livestock; *Coffea arabica* was used to treat LSD and pasteurellosis in bovines; abdominal pain and *aspiration pneumonia* of all livestock; *Coriandrum sativum* was used for bloat, abdominal pain and swelling in all livestock, including blackleg in bovines; *Erythrina brucei* was used for *aspiration pneumonia*, constipation, bloat and abdominal pain/ache in all livestock. *Euphorbia abyssinica* was used to treat asthma/stenosis of respiratory organs in equines; swelling, wound and tumour in all livestock; *Maesa lanceolata* to treat babesiosis*, aspiration pneumonia,* diarrhoea and rabies in all livestock; *Ocimum spicatum* was used to treat LSD in bovines; AHS in equines; eye pain in bovines; and michi in all livestock; *Rubia cordifolia* was used to treat *aspiration pneumonia* in bovines, sheep and goats; it was used to treat all livestock diarrhoea, bat urine ailment and michi*. Sida rhombifolia* was used to treat bloat and snake bite in all livestock; abdominal pain/ache and constipation in bovines, sheep and goats.

Thirteen plant species (9.85%) were used to treat three (0.92% each) ailments: *Aframomum corrorima* was used to treat abdominal pain, *aspiration pneumonia* and bloat in all livestock; *Aloe* sp was used to treat body swelling, diarrhoea and skin wound in all livestock; *Brucea antidysenterica* was used to treat blackleg of bovines; diarrhoea and bloat in all livestock; *Clematis longicauda* was used to treat pregnancy of bovines; babesiosis, and bloat in all livestock; *Clutia abyssinica* for *aspiration pneumonia* and toxicity of bovines, sheep and goats; actinomycosis of bovines; *Hesperocyparis lusitanica* was used to treat livestock trypanomiasis in bovines and equines; blackleg in bovines and dingetegna in all livestock.

*Foeniculum vulgare* was used to treat *aspiration pneumonia* of bovines, sheep and goats; blackleg and livestock TB of bovines; *Grevillea robusta* for LSD of bovines; constipation of bovines, sheep and goats; and bloat of all livestock. *Gymnanthemum* sp was used to treat dingetegna and diarrhoea of all livestock; livestock trypanomiasis of bovines and equines; *Prunus africanus* for swelling and bloat of all livestock; back sore of equines; *Ruta chalepensis* was used to treat abdominal pain/ache, bloat and trauma in all livestock; *Scepocarpus* *hypselodendron* was mainly used to treat constipation and livestock ascariasis in bovines, sheep and goats; bloat in all livestock; *Urtica simensis* was used to treat all livestock evil eye (evil spirit) and ailment of bat urine.

Of the 34 (25.76%) plant species, a single species was used to treat two livestock ailments (0.61% each), such as *Acmella caulirhiza* was used to treat the pregnancy (to initiate fertility) of bovines and bat urine ailment in all livestock; *Agave sisalana* swelling of all livestock and NCD of chickens; *Ajuga integrifolia* and *Asparagus africanus* were used to treat the evil eye and evil spirit of all livestock. *Allium cepa* was used to treat actinomycosis and livestock TB; *Artemisia absinthium* and *Cordia africana* were used effectively as livestock medicinal plants for *aspiration pneumonia* (severe coughing) in cattle, sheep and goats in addition of livestock trypanomiasis (bovines and equines), and bloat of all livestock, respectively. *Cymbopogon citratus* and *Gymnosporia arbutifolia* were also potential species used to treat eye pain (conjunctivitis) in all livestock separately including *aspiration pneumonia*, and *Brugmansia suaveolens* was used to treat diarrhoea and bloat in all livestock. *Carduus schimperi* was used for bovines pregnancy for foetus attachment; poisonous/toxicity plant eaten bovines, sheep and goats; *Bersama abyssinica* was used to treat anthrax and swelling of bovines, sheep and equines; swelling of all livestock; *Cyathula uncinulata* was used to treat *aspiration pneumonia* of bovines, sheep, and goats, and blackleg of bovines; *Cyphostemma adenocaule was* used to treat bovines body swelling and bloat of all livestock.

*Echinops kebericho* used to treat abdominal pain/ache and bloat; and *Euphorbia depauperata* to treat diarrhoea of all livestock and Lumpy Skin Disease of bovines; *Ilex mitis* was used to treat dingetegna and bloat; *Pentanema confertiflorum* was used to treat coughing and livestock TB in all livestock*. Justicia chimperiana* to treat diarrhoea in all livestock; FMD of the bovines*; Millettia ferruginea* was used to treat coughing and *spiration pneumonia* in all livestock; *Ocimum lamiifolium* for bloat and abdominal pain in all livestock; *Olinia rochetiana w*as used to treat *aspiration pneumonia* in all livestock and bovines pregnancy for foetus attachment*; Phaseolus lunatus* for dingetegna and diarrhoea in all livestock; and *Phytolacca dodecandra* was used to treat *aspiration pneumonia* and diarrhoea of all livestock*.*

*Ricinus communis* was used to treat eye pain and body swelling of all livestock; *Shirakiopsis elliptica* for diarrhoea and bat urine ailment in all livestock; *Scadoxus multiflorus* was used to treat bloat in all livestock and shoat pox in sheep and goats*; Vepris nobilis* and *Dodonaea viscosa* subsp. *angustifolia* were used to treat diarrhoea and bloat in all livestock; *Zanthoxylum asiaticum* was used to treat rabies and dingetegna in all livestock*; Gymnanthemum auriculiferum* was used to treat skin wound in all livestock and *fowl typhoid* in poultry; *Ximenia americana* was used to treat arthritis (gouge or mondy-morning ailment) of equines and Lumpy Skin Disease of bovines; *Phyllopentas schimperi* and *Coleus abyssinicus* were used to treat cysticercus (livestock ascariasis) of bovines, goats, and sheep; *Withania somnifera* wa*s* used mainly to treat arthritis of the equines and all livestock bloat.

Moreover, among the total reported plant species, 61 (46.21%) plants were used to treat one (0.31%) different ailment for instance *Achyranthes aspera*, *Albizia schimperiana, Crepis rueppellii, Rotheca myricoides, Lysimachia ruhmeriana, Olea welwitschii, Thymus schimperi,* and *Verbascum sinaiticum* were used to treat *aspiration pneumonia* more in bovines, sheep, and goats. *Antherica* sp*, Carissa spinarum, Helianthus annuus,* and *Kalanchoe hypseloleuce* were used to treat swelling in all livestock. *Apodytes dimidiata, Dicliptera foetida, Hagenia abyssinica* and *Pavetta oliveriana* were used to treat the ailment of bat urine or jaundice in all livestock.

*Oldeania alpina* was used to treat trauma (bone broken attachment) in bovines, sheep, and goats; *Balanites aegyptiaca* was used to treat arthritis (gouge) of equines*; Citrus x aurantiifolia* for actinomycosis of bovines; *Clematis hirsuta* was used to treat hepatitis of all livestock; *Colocasia esculenta* and *Lathyrus oleraceus* were used to treat nasal bote (parasitic leech) of all livestock. *Combretum molle, Myrtus communis* and *Platostoma africanum* to treat all livestock of diarrhoea; *Commelina benghalensis, Erica arborea, Ocimum basilicum* var. *cinnamon* and *Terminalia brownii* were used to treat constipation of bovines, sheep, and goats*; Lavandula dentata, Oncoba spinosa* and *Verbena officinalis* were used to treat abdominal pain/ache of bovines and sheep; *Melia azedarach* and *Physalis peruviana* were also used to treat dingetegna of all livestock*; Olea europaea* subsp. *cuspidata* was used to treat livestock tumour.

*Oxalis corniculata* was used to treat snake bite in all livestock; *Phyllopentas schimperi* was used to treat bovines, sheep and ascariasis of goats*. Peponium vogelii* was used to treat telleriosis of bovines and sheep. *Phoenix reclinata* and *Schrebera alata* were used to treat conjunctivitis (eye pain) in all livestock; *Rumex abyssinicus* was used to treat babesiosis in all livestock. *Rumex nepalensis* was used to treat livestock tumour (skin warts or venign external tumour) in bovines and equines; *Tapinanthus* sp of hemiparasite was used to treat LSD of bovines and equines, and *Trigonella foenum-graecum* was used to treat faciolosis of bovines, sheep, and goats*; Crinum abyssinicum* was used to treat skin wound of all livestock; *Cyperus rotundus*, *Erythrina abyssinica, Hordeum vulgare, Nigella sativa, Piliostigma thonningii, Rytigynia neglecta* and *Zea mays* were used to treat bloat of all livestock; *Datura stramonium*, *Ekebergia capensis*, *Afrocarpus falcatus,* and *Spiniluma oxyacantha* were used to treat all livestock aliment rabies*; Dicliptera magaliesbergensis* was used to treat reproduction problem of bovines; *Eragrostis tef* was used to treat sore of equines; *Lasiosiphon glaucus* was used to treat FMD of bovines*; Hymenodictyon floribundum* was used to treat livestock ascariasis of bovines*,* sheep, and goats*; Juniperus procera* was used to treat bovines actinobacillosis*.* All mentioned ethnoveterinary medicinal plants were used with different applications, preparation forms, means of treatment and roots of administration (Table 9) to treat dermatological, gastrointestinal, respiratory and other categorical different livestock ailments (Table 8).

In the study District, traditional healers diagnose livestock ailments before giving traditional medicines, mainly by observation, interviewing sick livestock owners and touching sick livestock body parts. During the diagnosis exercise, a traditional herbal practitioner identifies the ailment of a sick livestock; he or she has started well preparation and given the proper route and application. Moreover, herbal practitioners dealing with livestock ailments in the communities living in the study sites prepare remedies from different plants that play useful functions against infectious and non-infectious ailments.

### Toxicity/poisonous traditional livestock medicinal plants

From the study of livestock ailment-treating medicinal plant species *Calpurna aurea* (Fabaceae), *Datura stramonium* (Solanaceae), *Agarista salicifolia* (Ericaceae) and germinating *Sorghum bicolor* (Poaceae) at the growing stage with two leaves were also reported toxic plant species in addition to medicinal uses, and the traditional extracts of these ethnobotanical plants are used for various purposes. For example, informants reported that the traditional extract of *A. salicifolia* kills rats, and it also kills livestock when they eat the fresh leaves. *C. aurea* kills insects (repelling) and other livestock like mites, fleas and lice (for skin ailments). This species is said to be fata if swallowed and hence requires attention to neutralize its toxicity. Agricultural expansion, new settlements, local charcoal and overgrazing were the main threats to ethnoveterinary medicinal plants. These maximize the extinction of multi-purpose medicinal plants; attention was required for these poisonous species to neutralize toxicity.

#### Threats of ethnoveterinary medicinal plant species in the district

Plants in Soro District are threatened by different natural and anthropogenic factors as in many other districts. The major threats to medicinal plants identified by informants are deforestation due to the need for new farmlands for agricultural expansion and new settlements. Excessive use of shrubs and trees, from all habitats, for various functions. Overgrazing in the protected vegetation patches without awareness. The consequences of these activities lead to loss of vegetation, and decrease heritage of indigenous knowledge held by elders and the young generation would not have knowledge about use and management. These all-impact factors contribute to changes in climatic conditions to the environment which cause serious threats. Informed suggestions from different study sites for solutions of those different threats, to conserve those threatened medicinal plants either in the community or vegetation areas of the Soro District. Mainly in situ conservation in their natural habitats, teaching educational awareness in the community for the domestication of indigenous ethnoveterinary medicinal plants by local people around their lives, agricultural areas as shades, roadsides, nursery sites and reducing exotic substitutions. Hence, these help the sustainability of vegetation of the remained forest patches of the District.

## Discussion

In the study area, livestock are one of the main sources of the agricultural economy, providing power for crop production and livelihoods for the local community. In addition, mostly they provide various services for the rural community, including as pack animals, income sources, aspects of employment with survival values for human life. Furthermore, indigenous people in the local communities have different knowledge, practices and attitudes towards livestock medical healthcare. Besides, these ethnoveterinary ailments are controlled and prevented by various medicinal plant species, it is important to plan and apply implementation against specific ailments of livestock healthcare systems and their yield improvement [32–34]. The reason is that the owners of livestock and semi-pastoralists in the Soro District have rich indigenous knowledge and ethnoveterinary practices of herbal medicines and the use of medicinal plants for treating various specific livestock ailments. However, the inherited indigenous knowledge of the individuals varied with gender, age, literacy level, distance between humans and plants, knowledgeable and local informants, and agroecology.

The gender differences showed that males (about 65.89%) have rich information, inherited knowledge and healing practices on the use of livestock herbal medicinal plants on average (Table 3) compared with females (34.11%). This agrees with the findings reported from different parts of Ethiopia [6, 32, 35, 36]. In addition, Yirga et al. [37] also reported similar outcome information about 100% of herbal practitioners being males; similarly, 94.05% was reported by Yigezu et al. [35]. Abroad in China, the majority of the traditional livestock medicine practitioners relatively 56.7% were also dominated by males [38]. However, this result of the current study analysis, contrasting the findings of Yineger et al. [39], showed that female herbal practitioners were as knowledgeable as males. Elderly respondents (i.e. highly experienced older informants) quoted and knew more ethnoveterinary plant species on average than adults and youngsters; however, their differences were statistically insignificant (*P* > 0.05), and the findings did not agree with those of [32, 35], whereas educated informants shared and informed more knowledge on herbal medicine than the non-educated informants (Table 3), which was highly significant (*P* < 0.05) and might enhance the application of modern medicine practices due to written information rather than historical telling. These findings also disagree with those of the same authors. Similarly, there was a significant difference in medicinal plants reported from distantly rather than nearby to the main town. This is due to the fact that most of the informants were far from the veterinary healthcare centre, and they relied more on MPs to cure their livestock ailments; they were also highly significant among key respondents compared to general respondents. Because of this, more elder informants were involved in key informants than youngsters, which might increase the rate of local knowledge and biodiversity loss and stop the progress and continuity of knowledge from elders to future generations, likewise the study by Wondimu et al.[40]. Similarly, as observed, indigenous knowledge is disappearing due to most knowledgeable descendants dying without proper documentation of their knowledge, as reported by the same author. Even though a higher average number of medicinal plants were reported from Dega than from Woinadega and Kola, the difference was statistically not significant (*P* = 0.112; *P* ≥ 0.05); in this way, most informants can share indigenous knowledge information from different agroecologies.

In the various current study sites, the most informed herbalists were more men than women, with more herbal medicine practices that were related to various parts of Ethiopia [32, 33]; the reason is that most females take care of their children in their houses more than males; usually, they are culturally household owners with home activities more than males who work near and far from their living houses [35, 41], and shared more experienced information than females. In addition, males had opportunities to gain more enriching indigenous knowledge from their colleagues as well as the elders because they had the freedom to move from place to place. In addition to this, most experienced herbalists have the choice to transfer their indigenous knowledge to the preferable boy or mystery-holding girl when they approach death [42, 43]. The study also agrees with the result of [35], and it indicated that about 90% of livestock were treated by male owners rather than female owners with traditional medicines. Furthermore, another study also supports the output of this discovery [6, 8]. This finding refers to the unequal knowledge distribution among genders, which disagrees with the study of local knowledge of medicinal plants by [32], in the world, which stated that females gained and inherited more knowledge than males [44]; the differences are due to the culture of the society and the related intellectual achievements and interests in the various countries.

Different agroecological features, ages and vegetation differences in the study area contribute to diverse medicinal plants under varied agroecological and weather conditions. For example, from Shonkola kebele, a Mountain Shonkola forest patch of dry evergreen Afro-Montane Forest and Grassland complex vegetation types (DAF), the species *Agarista salicifolia*, *Oldeania alpina, Cordia africana,* *Erica arborea*, *Calpurnia aurea*, *Carissa spinarum*, *Clutia abyssinica*, *Euclea divinorum,* *Gymnosporia arbutifolia, Pentanema confertiflorum, Juniperus procera*, *Olea europaea* subsp*. cuspidata*, *Maesa lanceolata*, *Prunus africanus, Afrocarpus falcatus* and *Spiniluma oxyacantha* were some of the collected multipurpose medicinal indicator plant species; some species of *Combretum-Terminalia* vegetation types are *Combretum molle, Oncoba spinosa* and *Terminalia brownii*; from riverine plant species such as *Albizia schimperiana*, *Apodytes dimidiata, Croton macrostachyus*, *Erythrina abyssinica, Millettia ferruginea*, *Olea welwitschii, Olinia rochetiana, Phoenix reclinata, Vepris nobilis* and *Scherebra alata*; from the *Acacia-Commiphora* wood land forest patch area, plant species like *Balanites aegyptiaca, Piliostigma thonningii and Ximenia americana* were collected, where all representatives have various uses in the different local communities using different traditional knowledge in the three different agroecologies. Thus, the presence of this enormous traditional medicinal plant species in this current finding is highly important to sustain and continue indigenous knowledge with associated attributes, including multi-purpose plant species in natural habitats. Which prevent the loss of vegetation cover from the forest patches as well as elsewhere and increases the status of ethnoveterinary medical plant species, indigenous knowledge, herbal practitioners and wild edible plant species. The presence of this rich traditional knowledge and plant diversity among the people in the study area, the Soro people, could help to maintain and manage livestock health, food security and sovereignty, as well as conserve their foods for human beings against different livestock ailments. Although these knowledge practices are linked to the presence and sustainable continuity of ethnoveterinary traditional knowledge through the linkage of modern medicine and wild food plants with associated traditional knowledge for future generations. In the study area, the revitalizing lek linked to folk veterinary practices could be a concrete tool for promoting food sovereignty and traditional livestock healthcare, which can contribute to improving livestock health with food security. This also enhances livestock food security and provides many ecological transition advantages by increasing biodiversity and environmental balance.

Moreover, in developing countries, traditional medicine plant species have been indicated as being the most easily affordable and accessible to treat different types of veterinary ailments [45–47] and to use for economic purposes. The findings of the diverse use of ethnoveterinary plant species and the use of dominant families collected from high, mid and low lands were relatively comparable with the findings of other studies in different study areas of Africa, including Ethiopia and other world communities. For instance, in Ethiopia in the Dawuro Zone, Southern Ethiopia, local communities utilized, 103 EVMPs under 47 families for treatment of different LsAs; at the National Park of the Bale Mountains and adjacent areas, 74 medicinal and other multipurpose plants under 37 families were documented for treating 25 ailments [39]; in Sekota and Lalibela districts, 74 medicinal plants (MPs) under 31 families were also reported in four districts of Jimma Zone, Ethiopia, to treat 22 ailments [35], 53 medicinal plants under 31 families were documented for treating 22 different livestock ailments [48], with significant knowledge differences between gender, key and general participants, rural and urban inhabitants and informant age categories.

Information on ICFs, FL and PR values of documented medicinal plants would be necessary for future conservation priority species identification, antimicrobial activity and phytochemical studies, whereas direct matrix ranking exercise values are also useful impact factors to call urgent conservation attention to those locally threatened multipurpose livestock medicinal plants in the study area through anthropological activities [48].

Another study conducted in North Shewa, Ankober District 51 EVMPs under 35 botanical families and 50 genera to treat 33 different ailments were documented and published by [32], 49 EVMPs in Ada’a District of Afar Regional State to treat various livestock ailments [49], 48 EVMPs that belonging to 35 families used to treat 22 livestock health constraints in Dabo Hana District, in Western Ethiopia [50]. Similarly, other studies reported 34 LsMPs under 23 families for the treatment of 22 livestock ailments in Enarj Enawga District, East Gojjam Zone [6]. Moreover, the findings of the current study in Southern Ethiopia, Soro District Hadiya Zone documented relatively more varied numbers of livestock medicinal plants [132 LsMPs] under 61 families and 120 genera and associated indigenous knowledge to treat various veterinary ailments (about 50 ailments; Table 8) to prevent their impact on the livestock population. Also, varied agroclimatic plant species were reported, and the same medicinal plants were also used to control and treat different ailments in the three climatic conditions of the study sites. Some of the documented LsMP species in Soro District were similarly reported in other ethnoveterinary studies conducted in various parts of Ethiopia.

In this current study 132 of documented livestock medicinal plants, 29 species were reported by Temeche and Adeladlew [5] in the review status of ethnoveterinary medicine in Ethiopia; 29 species were reported in the National Park of the Bale Mountains and adjacent areas Yineger et al. [39]; 28 species by Yigezu et al. [35]; 25 species in Abergelle, Sekota and Lalibela districts of Amhara region, Northern Ethiopia by Assefa and Bahiru [48]; 24 species in selected Districts of Southern Ethiopia by Eshetu et al.[51]; 23 species from Ankober District, North Shewa Zone Amhara Region by Lulekal et al. [32]; 21 species from Ensaro District, North Shewa Zone [52]; 20 species from Wolmera District, Oromia Region [11]; 19 species by Tadesse and Dereje [4]; 16 species of Ethiopian medicinal plants for veterinary healthcare [9]; 15 species by Mesfin et al. [14]; 14 species from Leka Dullecha District, Western Ethiopia [53]; 13 species from Seharti-Samre District, Northern Ethiopia [7]; 12 species from Enarj Enawga District, East Gojjam Zone [6]; 11 species in the study of southern African medicinal plants [54]; nine species in South Wollo Zone [55]; six species in both Mojana Wodera District, Central Ethiopia [36] and Ada’ar District, Afar Region [49] were similarly well documented. These findings indicated that the widespread use of livestock medicinal plant species was indicated as LsMPs and associated local knowledge in preventing and controlling various veterinary ailments in different parts of Ethiopia. Furthermore, these traditional medicinal plant species are used to treat domestic livestock ailments in different geographical locations. It disseminates indigenous knowledge more widely across the community's geographical sites.

Some of the ethnoveterinary plant species were widely used and popular in the Soro District to treat various specific livestock ailments. For example, *Momordica foetida* was used to treat and manage diarrhoea, *aspiration pneumonia*, blackleg, anthrax, LSD, actinobacillosis (wooden tongue), and *Withinia somnifera* was used to treat and manage bloat, blackleg and arthritis (gouge or mondy-morning ailment). Similarly, other ethnoveterinary practitioners used *Withania somnifera* for the treatment of listeriosis and blackleg, which was also reported in different parts of Ethiopia, in Ada’ar District, Afar Region [49], and Ankober District, Amhara Region [56]. Another study, according to Tolossa et al.[57], also reported *Momordica foetida* ethnoveterinary medicinal use and management of blackleg in South Omo, Southern Ethiopia. This widespread use of the ethnomedicinal plant species in these different cultural groups of Ethiopia suggests their effectiveness in alleviating blackleg and deserves pharmacological investigations. *Cucumis ficifolius* was the other important recommended plant species used against anthrax, rabies, babesiosis, blackleg, dinegetegna and venign tumours in the study area. It agrees with the findings of other ethnoveterinary surveys conducted in different parts of Ethiopia, which witnessed the common use of *C. ficifolius* for the treatment of blackleg, according to Yigezu et al.[35], and rabies by Tadesse and Dereje [4]. Moreover, *Nicotiana tabacum* was used in the management of nasal bote/leech, snake bite and insects’ infection infestations in Soro District. Similarly, in Libo Kemkem District of the Amhara Region, Chekole et al.[58] suggested in a similar way the use of *N. tabacum* to treat leech infection, which agrees with Teklay et al.[59] in Kilte Awulaelo District, Tigray Region, using leech.

The majority of the rich ethnomedicinal plants were collected and reported from wild habitats (79.54%), which relatively agreed with the findings of the wild plant sources (78.79%) reported by Abebe [36] and 81.08% by Yigezu et al. [35], also, some were collected from agricultural croplands, and another few plant species such as *Antherica* sp, *Asparagus africanu*s and *Securidaca longepedunculata* were reported from the market survey in Soro District that were highly commercialized for the purpose of livestock medicines. In contrast in other parts of Ethiopia, *Embelia schimperi* and *Withania somnifera* were documented marketable plant species in the local markets of the Ankober District, North Shewa [32]. In addition, some numbers of food, food spices and condiments were reported from market survey of Soro District, plant spices, of these *Trigonella foenum-graecum* sold in the findings of Teklay et al. [59] in Kilte Awulaelo District, Tigray Region State reported similar species from a market survey that were sold as sources of food and spice. Moreover, a few others, indirectly from other ethnobotanical uses, like *Artemisia absinth*ium mainly sold by women, whereas *Nicotiana tabacum* sold from other social drug smoking in the market Jajura market by men traders. Moreover, our analysed results data of Soro District showed that Asteraceae ranked 1st, Fabaceae and Lamiaceae (2nd), Solanaceae (4th), Rubiaceae (5th), both Euphorbiaceae and Poaceae (6th), Amaryllidaceae and Rutaceae (8th) were dominant and frequently reported medicinal plant families (Fig. 2) and share livestock medicinal values in the country; in addition, in the world. The study similarly reported that Asteraceae, Lamiaceae and Euphorbiaceae were dominant families in Ethiopia which similarly studied by another investigators [6, 35, 39, 52, 60, 61]. Similarly, Asteraceae, Fabaceae and Solanaceae were dominant families in the study [36]. Whereas Asteraceae was the most commonly used and diversified medicinal plant family, which in line with the findings of [11, 14, 32, 34], it was one of the world leading largest families.

In Soro District, herbs were dominant finding and used for various medicinal preparation purposes by indigenous people, followed by shurbs (Fig. 1) and similarly reported by [6, 39, 61], and this information might be important for the survival of shrub and tree species from excessive harvesting. In addition, leaves (43.18%) were the most frequently utilized, preferable, easily available and simplicity in remedy preparation, and dominant harvesting plant parts in the current study for livestock medicinal use, and which agrees with many other studies in Ethiopia [11, 32, 35, 36, 48, 61–64] and also in South Africa [65]. Moreover medicinal practitioners use this highly available leaf part rather than root and bark parts to decrease the loss of plants from natural habitats [39, 66, 67].

In the current study most herbal medicine preparations were done mainly by decoction, concoction and crushing (Fig. 5); many livestock medicinal local practitioners used fresh plant parts to heal effectively and efficiently, mainly in the form of decoction using a single species followed concoction using two or more medicinal plant species to treat a single ailment, and this was disagreed by [11, 39], and agreed with study of [40]. In many sites of the study area, like several study areas in Ethiopia, medicinal preparation for use in different applications (Fig. 6) using fresh plant medicinal parts in combination or alone was documented. In addition, those dominantly useable medicinal fresh organs might be retained secondary bioactive metabolites that occurred more in fresh parts than in dry matters [32, 39, 61, 68, 69]. In the current study most herbal medicine preparations were done mainly by mixing a single, with two and more medicinal plant parts (Fig. 2) to treat a single ailment with cold and warm water, and using other locally available types of additives or without additives, which was similarly reported in other study parts [70, 71]. The oral treatment route is the main route of remedy administration in the study area (Fig. 3) and agrees with finding of other study parts of Ethiopia [5, 6, 39, 48, 49, 62, 70, 72], followed with dermal treatment which also agreed with [5, 6, 39] in common.

Informant consensus factor (ICF = 0.72, 0.71, and 0.70) showed the most prevalent ailments in each category in the study area and the least prevalent ailments with smaller ICF values had effective healing potential plant species (Table 4). Similarly, Lulekal et al. [32] reported a high informant consensus factor (0.71) to treat gastrointestinal ailments with popular curative plants. According to Sharma et al. [73] also similarly have shared high ICF for dermatological ailments that have a high incidence of livestock ailments and are treated using high curative potential plant species. Since values of high ICF are indicative of the selection of target plant species for the sake of future therapeutic drugs and other useful photochemical compounds [29]. Likewise in the Soro District, curative potential plants were used to treat the most prevalent livestock dermatological ailments for instance Foot and Mouth Disease/Ailment and Lumpy Skin Disease/Ailment were treated using *Croton macrostachyus,* blackleg treated by *Cyathula uncinulata* and ailment of bat urine by *Momordica foetida*; among gastro-intestinal potential ailment, diarrhoea, abdominal pain, acidiosis, anthrax, actinobacillosis (wooden tongue), telleriosis and New Castle Disease/Ailment (NCD/A) were treated by different healing effective plants discussed on preference ranking and FL values as well as a series respiratory infection like *aspiration pneumonia*, pasteurellosis (livestock TB) treated by *Pentanema confertiflorum* and *Stephania abyssin*ica. According to Lulekal et al. [32] plants with higher informant consensus values are thought to have more secondary bioactive metabolites for frequently occurring livestock ailments. These ranking activities showed that indigenous people highly depend on ethnoveterinary medicinal plants, even though the distribution of modern healthcare systems is rapidly increasing. The highest FL values (Table 5) among curative medicinal plants were accounted for *Datura stramonium* (100%) is highly effective to treat rabies from the neurological ailment, *Dodonaea viscosa* subsp. *angustifolia* (100%) in treating gastrointestinal PPR, *A.africanus* (100%) to treat evil eye/evil spirit; *Croton macrostachyus* (98%) for FMD among dermatological ailments, which is in line with the studies of [32, 35, 74]. Moreover, it indicated that *Croton macrostachyus* has the highest dominantly reported healing potential of plant species to treat dermatological ailments, and it was used to treat a variety of ailments alone or with combinations of other medicinal plants and additives in the study area. Others medicinal plant species *Albizia schimperiana* (96%) was used to treat *aspiration pneumonia*, and *Brugmansia suaveolens* to treat diarrhoea (92%), and they were the most important potential medicinal plants reported in the study area. FL is an important botanical tool to measure potential medicinal plants with the healing ability of the individual plant species and provide good information for future pharmacological investigation techniques, and it supported by [75]. Also, in preference ranking exercise (Table 6), *C. macrostachyus* also reported the highest (86%) and most efficacious to treatment Lumpy Skin Disease/Ailment followed *Ximenia americana* (75%) and *Allium sativum* (74%). In the study area, there were significant knowledge differences in ethno-therapeutic practices to protect livestock health between males and females, key and general participants, rural and urban inhabitants, and different age groups of informants (Table 3). Information on ICF, FL, and PR values of documented ethnoveterinary medicinal plants would be necessary for future antimicrobial activity and phytochemical studies, whereas DMR exercises (Table 7) also call for urgent conservation attention to those locally or nationally threatening multipurpose livestock medicinal plants in the study area through anthropological activities.

## Conclusion

This study showed that Soro District has diverse traditional medicinal plants used for treating various livestock ailments, using indigenous and local ethnoveterinary knowledge, and ethnoveterinary skills and practices. In this investigation, 132 ethnoveterinary medicinal plants were documented to treat 50 livestock ailments. The data on medicinal plant species were collected, confirmed and documented from different study sites (*n* = 13) in the District that help to defend against various types of potential livestock ailments and are used for various functions. Most of them were used to treat a single livestock ailment; others were used to treat poly-ailments with different plant parts prepared alone or poly-medicinal plants with the use of other additives or without additives. Through the study in the selected kebeles, *Carduus schimperi* and *Clutia abyssinica* were cited for use as antidotes in the event of severe reactions of poisonous plant species eaten by livestock. In addition, they are also important medicinal plant species. The majority of the medicinal plants were reported from the natural wild habitats in different agroecological areas. Some were reported in the localities of agricultural lands and stalling vendors of an open local market, for example, *Antherica* sp., *A. africanus, Securidaca longepedunculata,* and some others were from market-stalling sites for spices that were sold as food, food flavours or indirectly sold by women for medicinal uses that were brought from their rich homegardens, commonly *Artemisia absinthium*, *Allium sativum, Coriandrum sativum, Foeniculum vulgare, Ocimum basilicum,* and *Ruta chalepensis*. Among food products: *Ensete ventricosum*, *Eragrostis tef*, *Hordeum vulgare* and *Zea mays*; vegetable foods of *Colocasia esculenta*; and stimulants: *Coffea arabica* and *Nicotiana tabacum.* Moreover, knowledge use in medicinal plants exists with significant differences among parameters. Ethnobotanical tools (ICF, FL and PR) provided good information for setting more conservation priorities, remedy utilization and future anti-microbial activities on claimed highest-ranked potential curative medicinal plant species, making them more essential inputs for future therapeutic drug inquiries to develop modern medicines. DMR exercises on use attributes hinted at the need for setting up conservation priority for plant species such as *Prunus africana*, *Combretum molle, Afrocarpus falcatus* and *Olea welwitschii*, and species reported in preference ranking (PR) from homegardens *A. sativum* and *Croton macrostachyus* in woinadega*, Ximenia americana* in kola, and *Juniperus procera* more from dega agroecology. In the FL, *Datura stramonium* against rabies*, D. angustifolia* against PPR*, A. africanus* against evil eye (evil spirit, including other ailments) and *C. macrostachyus* efficacy to treat FMD and potentially promising species with respect to others treating different livestock ailments were recoded*.* These important ethnoveterinary plants were found under various threats as a result of various anthropological and environmental factors, and hence conservation attention is required to prevent the decline of these flora. Also, it calls for researchers to raise awareness with the consultation of community-targeted traditional practitioners, including agriculturalists and ethnobotanists, and ecologists to adopt lifestyles focussed to sustainable use. Therefore, this would enrich and save diverse multipurpose medicinal plants with associated indigenous herbal knowledge in the study area. Most of the identified and confirmed ethnoveterinary plant species in the current study could be effective for future phytochemical and pharmacological activities, and they have also warranted the future profile of the plant species reported by indigenous people.

## Data Availability

All the data used to support the findings of this manuscript are available in this paper.

## Abbreviations

AAU: Addis Ababa University
ANOVA: Analytical variance analysis
Bsc: Bachelor of Science in Veterinary Nursing
DMR: Direct matrix ranking
DVM: Doctor of Veterinary Medicine
EPHI: Ethiopian Public Health Institute
EVMPs: Ethnoveterinary medicinal plants
FGDs: Focus group discussions
FL: Index of fidelity level
GPS: Geographic Positioning System
ICF: Informant consensus factor
ICPC: International Classification of Primary Care
Livestock TB: Livestock tuberculosis
LsAs: Livestock ailments
LSD: Lumpy Skin Disease
LsMPs: Livestock medicinal plants
NCD: New Castle Disease
PPR: Peste des petits ruminants
PR: Preference ranking
SD: Standard deviation
SPSS: Statistical Package for Social Science
